# rWTC‐MBTA Vaccine Induces Potent Adaptive Immune Responses Against Glioblastomas via Dynamic Activation of Dendritic Cells

**DOI:** 10.1002/advs.202308280

**Published:** 2024-01-31

**Authors:** Herui Wang, Rogelio Medina, Juan Ye, Yaping Zhang, Samik Chakraborty, Alex Valenzuela, Ondrej Uher, Katerina Hadrava Vanova, Mitchell Sun, Xueyu Sang, Deric M. Park, Jan Zenka, Mark R. Gilbert, Karel Pacak, Zhengping Zhuang

**Affiliations:** ^1^ Neuro‐Oncology Branch National Cancer Institute National Institutes of Health Bethesda Maryland 10022 USA; ^2^ NE1 Inc. New York NY 10022 USA; ^3^ Eunice Kennedy Shriver National Institute of Child Health and Human Development National Institutes of Health 9000 Rockville Pike Bethesda MD 20892 USA; ^4^ John Theurer Cancer Center HUMC Hackensack Meridian School of Medicine 92 2nd St Hackensack NJ 07601 USA; ^5^ Department of Medical Biology Faculty of Science University of South Bohemia České Budějovice 37005 Czech Republic; ^6^ Present address: Staff Scientist Neuro‐Oncology Branch National Cancer Institute Center for Cancer Research National Institutes of Health Building 37 Room 100437 Convent Dr. Bethesda MD 20892 USA; ^7^ Present address: Senior Investigator Neuro‐Oncology Branch National Cancer Institute Center for Cancer Research National Institutes of Health Building 37 Room 100037 Convent Dr Bethesda MD 20892 USA

**Keywords:** dendritic cells, glioblastoma, immunotherapy, rWTC‐MBTA vaccine, tumor microenvironment

## Abstract

Despite strides in immunotherapy, glioblastoma multiforme (GBM) remains challenging due to low inherent immunogenicity and suppressive tumor microenvironment. Converting “cold” GBMs to “hot” is crucial for immune activation and improved outcomes. This study comprehensively characterized a therapeutic vaccination strategy for preclinical GBM models. The vaccine consists of Mannan‐BAM‐anchored irradiated whole tumor cells, Toll‐like receptor ligands [lipoteichoic acid (LTA), polyinosinic‐polycytidylic acid (Poly (I:C)), and resiquimod (R‐848)], and anti‐CD40 agonistic antibody (rWTC‐MBTA). Intracranial GBM models (GL261, SB28 cells) are used to evaluate the vaccine efficacy. A substantial number of vaccinated mice exhibited complete regression of GBM tumors in a T‐cell‐dependent manner, with no significant toxicity. Long‐term tumor‐specific immune memory is confirmed upon tumor rechallenge. In the vaccine‐draining lymph nodes of the SB28 model, rWTC‐MBTA vaccination triggered a major rise in conventional dendritic cell type 1 (cDC1) 12 h post‐treatment, followed by an increase in conventional dendritic cell type 2 (cDC2), monocyte‐derived dendritic cell (moDC), and plasmacytoid dendritic cell (pDC) on Day 5 and Day 13. Enhanced cytotoxicity of CD4+ and CD8+ T cells in vaccinated mice is verified in co‐culture with tumor cells. Analyses of immunosuppressive signals (T‐cell exhaustion, myeloid‐derived suppressor cells (MDSC), M2 macrophages) in the GBM microenvironment suggest potential combinations with other immunotherapies for enhanced efficacy. In conclusion, the authors findings demonstrate that rWTC‐MBTA induces potent and long‐term adaptive immune responses against GBM.

## Graphical abstract

Representative histology images of brain tumor areas in the control group and vaccine (rGL261‐MBTA) group on day 13 after the start of treatments. Small magnification. Scale bars, 600 µm. High magnification of dashed rectangle area. Scale bar, 60 µm. High magnification of indicated dashed rectangle areas. Red arrows indicate infiltrating lymphocytes. Scale bars, 60 µm.

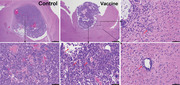

## 1 Introduction

Glioblastoma multiforme (GBM) is the most common and aggressive brain malignancy in adults.^[^ ^1^ ^]^ GBM poses a formidable challenge with the current standard of care for newly diagnosed GBM involving maximal surgical resection, followed by radiotherapy and temozolomide.^[^ ^2^ ^]^ Despite this treatment, the median overall survival for GBM patients remains less than 15 months, underscoring the urgent need for novel therapeutics.^[^ ^2^ ^]^

Among the arsenal of cancer immunotherapies, checkpoint inhibition through PD‐1 (e.g., Cemiplimab, Nivolumab, and Pembrolizumab), PD‐L1 (e.g., Atezolizumab, Avelumab, Durvalumab), and CTLA‐4 (Ipilimumab) have shown promising efficacy against multiple metastatic cancers, including breast, melanoma, and lung cancers.^[^ ^3^, ^4^ ^]^ However, the translation of checkpoint inhibitors to GBM was only effective in patients with mismatch repair deficiency.^[^ ^5^ ^]^ Consequently, other immunotherapies, including chimeric antigen receptor‐modified T (CAR‐T) cells, have not had much success in GBM clinical trials.^[^ ^6^ ^]^ The challenges faced in treating GBM are often rooted in the tumor's characterization as an immunogenically “cold” tumor due to its inherently low immunogenicity and the presence of an immunosuppressive tumor microenvironment (TME). The TME is marked by abundant immunosuppressive cells and factors, including tumor‐associated macrophages (TAMs), myeloid‐derived suppressor cells (MDSCs), regulatory T cells (Tregs), interleukin‐10 (IL‐10), and transforming growth factor ß (TGF‐ß), which are unfavorable for the recruitment and infiltration of effector T cells.^[^ ^7^, ^8^, ^9^, ^10^ ^]^ Thus, converting “cold” tumors into “hot” ones is pivotal for enhancing immune activation and improving treatment outcomes.^[^ ^9^ ^]^

Concurrently, cancer vaccines have emerged as a promising therapeutic platform in juxtaposition to conventional immunotherapies as they stimulate the host's immune system to generate tumor‐specific immune responses for tumor recognition and elimination.^[^ ^11^ ^]^ While initial therapies aimed to target specific tumor‐associated antigens, the high intratumoral heterogeneity of GBMs makes it difficult to identify epitopes that are tumor‐specific and ubiquitously expressed. Instead of specific epitopes, whole tumor lysate is becoming a promising clinical approach because of its ability to present a repertoire of tumor antigens to a patient's immune system for the selection and expansion of multiple tumor‐specific lymphocyte clones.^[^ ^12^ ^]^ From this hypothesis, a Phase 3 clinical trial (NCT00045968) has been launched describing an autologous tumor lysate‐pulsed dendritic cell vaccine (DCVax‐L) in newly diagnosed and recurrent GBMs. The clinical trial results indicate that adding DCVax‐L to standard therapy is feasible and safe in GBM patients and may extend their survival.^[^ ^13^, ^14^ ^]^ However, these vaccines face several hurdles including time and labor‐intensive production, limited migration, and overcoming the immunosuppressive TME.^[^ ^15^ ^]^

Dendritic cells (DCs) play a central role in cancer vaccination. They are major coordinators between innate immunity and adaptive immunity. Based on the ontogeny studies and gene expression profiling, dendritic cells are divided into conventional DC (cDC), plasmacytoid DC (pDC), monocyte‐derived DC (moDC), and Langerhans cell (LC).^[^ ^16^, ^17^ ^]^ LCs are considered a specialized subset of the tissue‐resident macrophages.^[^ ^18^ ^]^ cDCs, also known as classical DCs, are the major DCs that prime naïve T cells and restimulate memory T cells in secondary lymphoid tissues like the lymph node (LN).^[^ ^19^ ^]^ Type 1 cDCs (cDC1) are the primary subset to cross‐present antigens to CD8+ T cells, and type 2 cDCs (cDC2) are largely associated with CD4+ T cell responses.^[^ ^16^ ^]^ In preclinical GBM models, DC subsets (cDC1, cDC2, moDC, pDC) were able to phagocytose tumor antigens and migrate to cervical LN, and cDC1 was confirmed to prime CD8+ T cells and drive tumor‐specific CD8+ T cell responses against GBM.^[^ ^20^ ^]^ Nowadays, immunostimulant adjuvants are widely used in cancer vaccines to improve tumor antigen uptake by DCs, induce DC activation and migration, and stimulate inflammatory cytokine production.^[^ ^12^ ^]^

Our group recently developed a personalized cancer vaccine strategy leveraging multiple immunogenic components to trigger innate and adaptive immune responses against cancer cells.^[^ ^21^ ^]^ Our approach involves irradiated whole tumor cells (rWTC) pulsed with a combination of mannan‐BAM, Toll‐like receptor (TLR) agonists [lipoteichoic acid (LTA), polyinosinic‐polycytidylic acid (poly (I:C)), and resiquimod (R‐848)], and anti‐CD40 monoclonal antibody (rWTC‐MBTA).^[^ ^21^ ^]^ This vaccine strategy inhibited tumor growth in the CT26‐colon carcinoma model, an immunologically “hot” tumor model,^[^ ^21^, ^22^ ^]^ and also prevented early metastasis in preclinical models.^[^ ^23^ ^]^ However, it is still unknown whether this strategy can treat immunologically “cold” tumors such as GBM, considering the specialized immune microenvironment in the brain.^[^ ^24^ ^]^ Also, the detailed effects of the rWTC‐MBTA vaccine on DC subsets and TME have not been well characterized.

In the present study, we investigated the therapeutic potential of the rWTC‐MBTA vaccine strategy in murine GBM models. We demonstrate that rWTC‐MBTA dynamically activated DC subsets, triggered potent adaptive immune responses against GBM, and established long‐term immune memory. Analysis of the TME also proposes compelling opportunities for synergistic combinations with other immunotherapies.

## 2 Results

### 2.1 rWTC‐MBTA Vaccine Significantly Prolongs Survival and Eliminates GBM in the GL261 Animal Model

**Figure 1:**
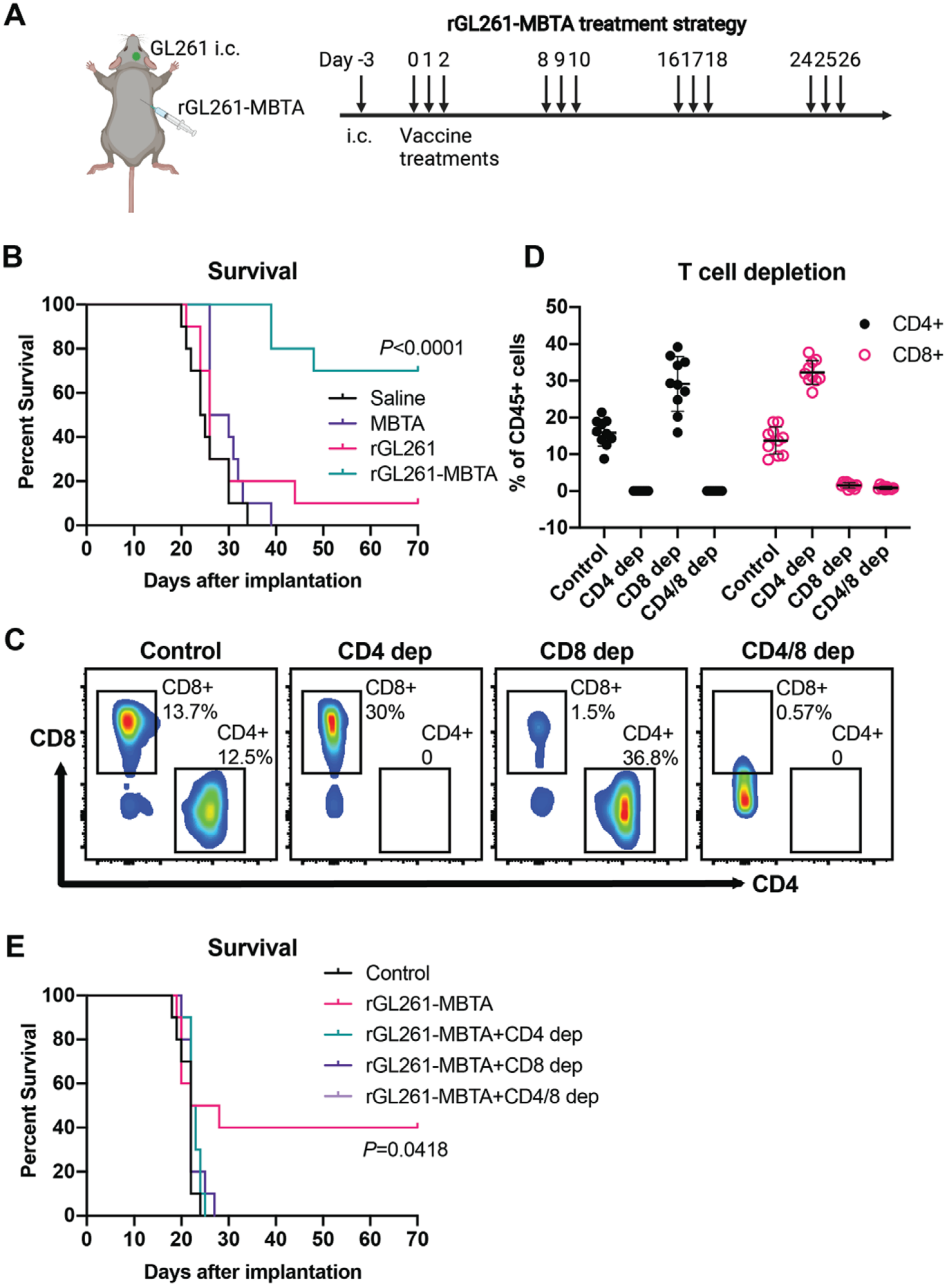
rGL261‐MBTA vaccine significantly prolongs survival of the GL261 GBM model, which depends on the adaptive immune response. A) Treatment strategy of rGL261‐MBTA vaccine. The GL261 GBM model was established by intracranial injection of 50000 GL261 cells. Three days after tumor cell implantation, the rGL261‐MBTA vaccine or other indicated treatments (saline control, MBTA only, rGL261) were injected subcutaneously into the right flank region. The treatments last for four weeks, with three consecutive injections per week. Arrows labeled the injection days. B) Kaplan‐Meier survival curves of different treatment groups. N = 10 for each group. *P*<0.0001, Log‐rank test. C) Representative flow cytometry results of CD4+ and CD8+ T cells in peripheral blood of control and T cell depletion mice. Dep, depletion. CD4+T% and CD8+T% of total CD45+ cells were labeled next to the gated population. D) Summary of CD4+T% and CD8+T% of whole CD45+ cells in peripheral blood confirmed robust depletion of CD4+T and CD8+T cells in indicated depletion groups. N = 10 for each group. E) Kaplan‐Meier survival curves of rGL261‐MBTA treated control and T cell depletion groups. The saline control group was also included. N = 10 for each group. *P* = 0.0418, Log‐rank test for trend.

To test the hypothesis that the rWTC‐MBTA vaccine generates an adaptive immune response and improves the survival of GBM‐bearing mice, we established an intracranial murine glioma model with GL261 cells. C57BL/6 mice were inoculated in the right frontal lobe with syngeneic GL261 cells (50000 each) and, after three days, were randomly divided into four groups: normal saline (control), MBTA only, irradiated GL261 cells (rGL261) and rGL261‐MBTA (irradiated GL261 cells mixed with MBTA). rGL261‐MBTA vaccine and other treatments were administered to the right flank every day for three days and then repeated weekly for a total of 4 weeks (**Figure** **1**A). All mice were monitored until the study endpoint.

Mice in the rGL261‐MBTA vaccine treatment group demonstrated a significant increase in survival compared to saline, MBTA, and rGL261‐treated mice (*P*<0.0001, Log‐rank test) (Figure 1B). In contrast, mice in the MBTA and rGL261 treatment groups demonstrated no significant increase in survival compared to saline‐treated control mice. However, one out of ten (10%) of rGL261‐treated mice achieved complete remission (CR) in this study, indicating that irradiated whole tumor cells alone may contribute to mild antitumor immunity, consistent with previous reports.^[^ ^12^ ^]^ Of note, 7 out of 10 (70%) of rGL261‐MBTA vaccine‐treated mice achieved CR for the duration of the study (Figure 1B). These results suggest that treatment with twelve rGL261‐MBTA vaccines successfully generated a potent antitumor response against intracranial GL261 tumors.

To assess if the observed antitumor effects depended on T cells, GL261 tumor‐bearing mice were subjected to CD4+, CD8+, or CD4+ and CD8+ (CD4+/CD8+) T cell depletion. CD4+, CD8+, and CD4+/CD8+ T‐cell‐depleted mice were subjected to the same rGL261‐MBTA vaccine treatment. Robust CD4+ and CD8+ T‐cell depletion in peripheral blood samples of indicated groups was confirmed via flow cytometry staining seven days after initiating tumor vaccine treatment (Figure 1C,D). CD4+, CD8+, and CD4+/CD8+ T‐cell‐depleted rGL261‐MBTA vaccinated mice demonstrated no increase in survival when compared to saline‐treated control mice (Figure 1E). In contrast, 4 out of 10 normal rGL261‐MBTA treated mice achieved CR (Figure 1E), suggesting that the rGL261‐MBTA vaccine's therapeutic efficacy against GL261 tumors depends on both CD4+ and CD8+ T cell‐mediated immune responses.

### 2.2 rGL261‐MBTA Vaccine Activates DCs in Local Draining LNs

To assess the mechanism of action of the rGL261‐MBTA vaccine, we first investigated if the rGL261‐MBTA vaccine augmented the trafficking of antigen‐presenting cells (APCs) to local draining lymph nodes that are close to the subcutaneous vaccination site. Thirteen days after the start of tumor vaccine treatments (mice had already received a total of six doses of tumor vaccination or other treatments), a flow panel of innate immune cells showed that the rGL261‐MBTA vaccine increased APC populations in local LNs compared to saline control mice. Of note, DCs were significantly augmented in rGL261‐MBTA vaccinated mice [(1.48±0.3)% of total CD45+ cells, mean±SD, same below] compared to the control group [(0.67±0.12)%] (Supp. Figure 1A). rGL261‐MBTA vaccinated mice also demonstrated significant increases in other types of APCs compared to saline control mice, such as MHCII+ monocytes [(0.33±0.12)% versus (0.03±0.02)%] and macrophages [(0.26±0.12)% versus (0.16±0.06)%] (Supp. Figure 1B‐D). It's noteworthy that the MBTA treatment group also demonstrated a significant increase in DCs [(1.59±0.76)%] and MHCII+ monocytes [(0.21±0.1)%], compared to the control group (Figure S1, Supporting Information). This result indicates that MBTA components (Mannan‐BAM, TLR agonists, anti‐CD40 antibody) are sufficient to activate these innate immune cells. However, the absence of irradiated tumor cells as the tumor antigen source prevents the activated APCs from inducing a tumor‐specific adaptive immune response in MBTA‐treated mice.

**Figure 2:**
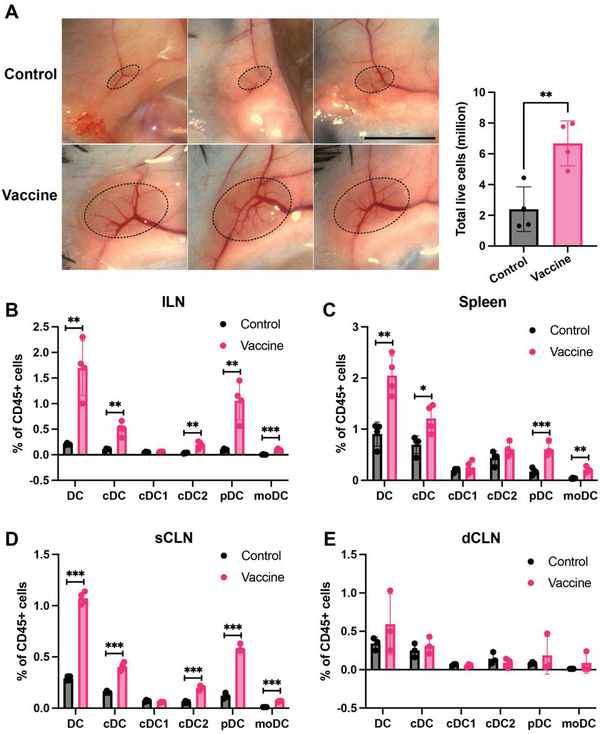
rGL261‐MBTA increased DC subtype populations in the peripheral lymph nodes and spleen on day 13 from the start of the vaccine treatment. A) Representative inguinal lymph node (ILN) for control and vaccine mice. ILN is outlined by dashed lines. ILN cell counts after dissociation are presented on the right side. N = 4 for each group. **, *P*<0.01, unpaired t test. Vaccine, rGL261‐MBTA treatment group. B–E) The percentage of DC, cDC, cDC1, cDC2, pDC, and moDC of total CD45+ cells in ILN (B), spleen (C), sCLN (D), and dCLN (E). DC, dendritic cells; cDC, conventional DC; cDC1, cDC type 1; cDC2, cDC type 2; pDC, plasmacytoid DC; moDC, monocyte‐derived DC; sCLN, superficial cervical lymph node; dCLN, deep cervical lymph node. N = 4 for each group. *, *P*<0.05; **, *P*<0.01; ***, *P*<0.001; unpaired t test.

We then focused on DCs and characterized DC subsets in the LNs and spleen. We confirmed enlarged inguinal lymph nodes (ILNs) and spleens on Day 7 and Day 13 from the start of the vaccination (Figure S2A, Supporting Information and **Figure** **2**A). Compared to the control group on Day 13, out of the total CD45+ cells, the rGL261‐MBTA vaccine group exhibited increased percentages of cDC, pDC and moDC in ILNs, superficial cervical LNs (sCLNs), and spleens (Figure 2B,D), confirming the extensive effects of the vaccination on DC subsets. We also checked the subtypes of cDC and confirmed no significant change in the percentage of cDC1 but a significant increase in the percentage of cDC2 in ILN and sCLN (Figure 2B,D). In contrast, we did not observe a significant change in DC subsets in the deep cervical LNs (dCLNs) (Figure 2E). Considering the size difference of the ILN and spleens between the control and vaccine (rGL261‐MBTA) groups, we also compared the absolute number of DC subsets in these tissues and confirmed the consistent increase in the vaccine group (Figure S2B,C, Supporting Information).

### 2.3 rGL261‐MBTA Induces a Potent Adaptive Immune Response

**Figure 3:**
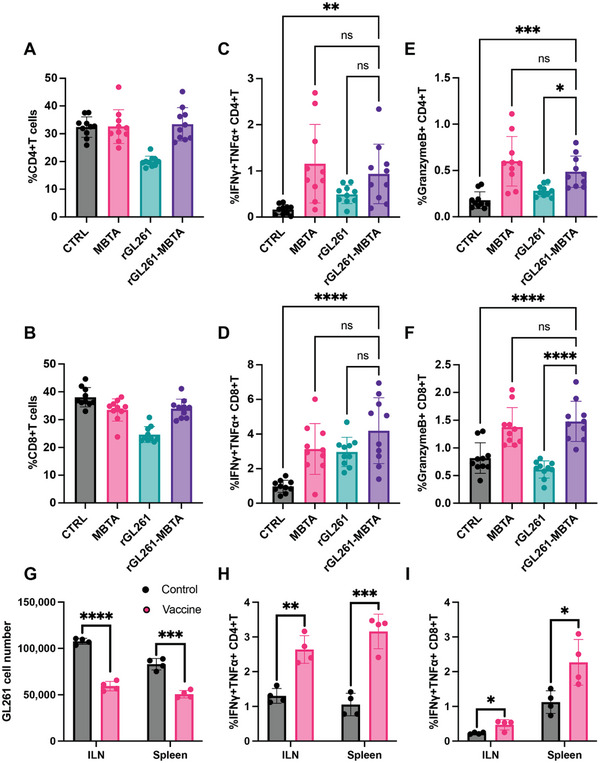
rGL261‐MBTA vaccine enhanced adaptive immunity in draining lymph nodes. A) %CD4+T cells of CD45+ cells in branchial lymph nodes. N = 10 for each group. CTRL, control group, same elsewhere. B) %CD8+T cells of CD45+ cells in branchial lymph nodes. N = 10 for each group. C,D) The percentage of IFNγ+TNFα+ CD4+T (C) and CD8+T cells (D) in CD4+T and CD8+T cells, respectively, in branchial lymph nodes after PMA activation. N = 10 for each group. ns, *P*>0.05; **, *P*<0.01; ****, *P*<0.0001; One‐way ANOVA with multiple comparisons. E,F) Granzyme B+ CD4+T (E) and CD8+T cells (F) percentage of CD4+T and CD8+T cells, respectively, in branchial lymph nodes after PMA activation. N = 10 for each group. ns, *P*>0.05; *, *P*<0.05; ***, *P*<0.001; ****, *P*<0.0001; One‐way ANOVA with multiple comparisons. (G) Live GL261 tumor cell counts after co‐culture with indicated dissociated cells. ILN, inguinal lymph node. Red columns indicate the vaccine group (rGL261‐MBTA). N = 4 for each group. ***, *P*<0.001; ****, *P*<0.0001; unpaired t test. H,I) IFNγ+TNFα+ CD4+T (H) and CD8+T cells (I) percentage after co‐culture with GL261 tumor cells. Red columns indicate the vaccine group (rGL261‐MBTA). N = 4 for each group. *, *P*<0.05; **, *P*<0.01; ***, *P*<0.001; unpaired t test.

Next, we evaluated if the rGL261‐MBTA vaccine could activate an adaptive immune response against GL261 cells in isolated LNs and brain TME. In the LNs, rGL261‐MBTA vaccinated mice did not reveal obvious changes in the percentage of CD4+ or CD8+ T cell populations of total CD45+ cells compared to saline control mice (**Figure** **3**A,B). However, further investigations into the functional states of CD4+ and CD8+ T cells of rGL261‐MBTA treated mice after 5‐hour PMA (Phorbol 12‐myristate 13‐acetate) activation revealed more cytotoxic signatures when compared to the saline control group (Figure 3C–F). Specifically, IFNγ+TNFα+ and Granzyme B+ CD4+T and CD8+T cells were all increased in the vaccinated mice, compared to the control group (Figure 3C–F). rGL261 alone showed weaker T cell activation than the rGL261‐MBTA vaccine group upon PMA treatment (Figure 3E,F), consistent with the weak immunogenicity of irradiated tumor cells previously reported.^[^ ^12^ ^]^ Notably, the MBTA alone group showed comparable cytotoxic signatures (IFNγ+TNFα+ and Granzyme B+) in the CD4 and CD8 T cells after PMA activation, compared to the rGL261‐MBTA vaccine group. However, these PMA‐activated T cells in the MBTA group are not expected to be tumor‐specific because the activated DCs were not able to present tumor antigens to the T cells without a source of tumor antigens.

To confirm the increased tumor‐specific adaptive immune response in the vaccinated mice, we co‐cultured the immune cells of ILN and spleen with GL261 tumor cells. Two days later, the live GL261 cell number in the vaccine co‐culture group was about half of that in the control group (Figure 3G). IFNγ+TNFα+ CD4+T and CD8+T cells were also increased in the vaccine co‐culture group (Figure 3H,I). These results confirmed that the rGL261‐MBTA vaccine stimulated tumor‐specific immunity in the lymphoid organs.

### 2.4 rGL261‐MBTA Induces Potent Anti‐GL261 Effects in the Brain

We then examined TME in GL261 tumors. Regarding the DCs infiltrating tumor tissues, we did not obtain a significant difference in the percentage of each DC subtype between control and vaccine groups (Figure S3A, Supporting Information). However, the immunostimulatory signals were increased in cDC and other DC subtypes (Figure S3B–E, Supporting Information). Specifically, the CD80 expression was increased in cDCs, including cDC1 and cDC2, and moDCs, and the CD86 expression was also increased in cDC and pDC cells (Figure S3B,C, Supporting Information).

**Figure 4:**
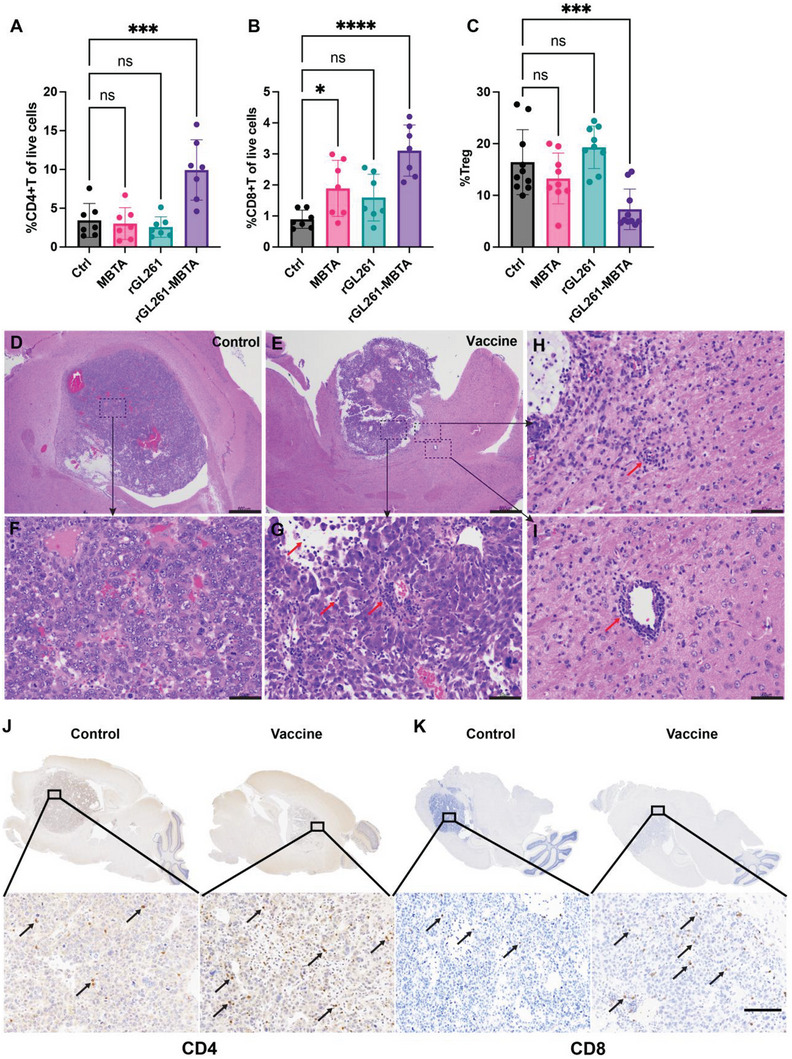
rGL261‐MBTA vaccine enhanced adaptive immunity in brain tumor areas. A,B) %CD4+Tcells (A) and %CD8+Tcells (B) of total live tumor dissociated cells in different treatment groups. N = 7 for each group. ns, *P*>0.05; *, *P*<0.05; ***, *P*<0.001; ****, *P*<0.0001; One‐way ANOVA with multiple comparisons. C) %Treg of whole CD4+T cells in each group. N = 10 for control and rGL261‐MBTA groups, respectively. N = 9 for MBTA and rGL261 groups, respectively. ns, *P*>0.05; ***, *P*<0.001; One‐way ANOVA with multiple comparisons. D,E) Representative histology images of brain tumor areas in the control group (D) and vaccine (rGL261‐MBTA) group (E) on day 13 after the start of treatments. Small magnification. Scale bars, 600 µm. F) High magnification of dashed rectangle area in D. Scale bar, 60 µm. G,I) High magnification of indicated dashed rectangle areas in E. Red arrows indicate infiltrating lymphocytes. Scale bars, 60 µm. J,K) Representative CD4 (J) and CD8 K) IHC staining of control and vaccinated tumors. Arrows indicate representative CD4+ and CD8+ T cells. N = 3 for the control group and N = 2 for the vaccine group. Scale bars, 100 µm.

We then examined the CD4+ and CD8+ T cells in the GL261 tumors. On Day 13, TIL analyses confirmed increased %CD4+T [(9.93±3.89)% versus (3.43±2.18)% of total live dissociated cells] and %CD8+T [(3.11±0.82)% versus (0.89±0.29)%] in the vaccinated tumors, compared to the control group (**Figure** **4**A,B). We also noticed that Tregs were decreased in the vaccinated mice [(7.31±3.92)% versus (16.43±6.29)%] (Figure 4C). We also collected GL261 brain tumors for histology examination. H&E staining revealed smaller tumor size and more tumor‐infiltrating lymphocytes in rGL261‐MBTA vaccine mice than saline control mice (Figure 4D–I). Notably, infiltrating lymphocytes were “chasing” the spreading tumor cells in the adjacent brain tissues in the rGL261‐MBTA vaccine group (Figure 4H,I). Immunohistochemistry (IHC) staining also confirmed increased CD4+ and CD8+ T cells in the tumor tissues of vaccinated mice compared with control tumor tissue (Figure 4J,K). CD4+ and CD8+ T cell distribution throughout the tumor area also indicated potent T cell infiltration in GL261 GBM models.

Because T cell exhaustion is a cornerstone of many immunotherapy failures,^[^ ^25^ ^]^ we checked our vaccine's impact on T‐cell states and the tumor immunosuppressive TME in the GBM tumors. We first quantified the exhaustion markers (CTLA4, PD1, LAG3, TIM3) in the CD4+ and CD8+ T cells after vaccination. On day 13, we did not observe obvious changes in these exhaustion markers between the control and vaccine groups, except for an increased TIM3+ CD8+ T cells in the vaccine group [(32.07±8.23)% versus (13.68±10.72)% in the control group] (Figure S4, Supporting Information). Interestingly, the MBTA alone group showed decreased LAG3+, LAG3+&PD1+, LAG3+&TIM3+, and LAG3+&PD1+&TIM3+ CD4+ T cells, while the rGL261 group showed increased CD8+ T cells expressing multiple exhaustion markers (LAG3+, TIM3+, LAG3+&PD1+, LAG3+&TIM3+, PD1+&TIM3+, and LAG3+&PD1+&TIM3+).

We then examined the immunosuppressive cells in the brain microenvironment. MDSCs consist of mononuclear MDSC (M‐MDSC) and polymorphonuclear MDSC (PMN‐MDSC). We observed significantly increased PMN‐MDSC in the rGL261 group and significantly decreased M‐MDSC and total MDSC in the MBTA alone group. However, with the combination of MBTA and rGL261 in the vaccine (rGL261‐MBTA) group, no significant differences in MDSC or its subgroups were obtained between the control and vaccine groups (Figure S5A–C, Supporting Information). We also examined the immunosuppressive M2 macrophages in the GBM tissues. Interestingly, the percentage of M2 macrophages was not changed in the vaccine group, but the PDL1 expression in M2 macrophages was significantly increased in the vaccine group (Figure S5D,E, Supporting Information).

### 2.5 rGL261‐MBTA Established Tumor‐Specific Immunity and Long‐Term Immunological Memory Against GL261 Tumor Cells

**Figure 5:**
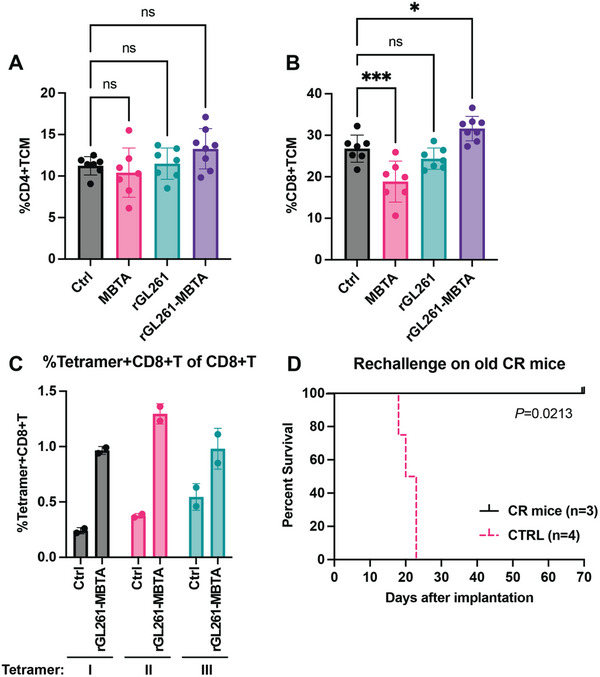
rGL261‐MBTA vaccine induced long‐term immune memory against GL261 tumors. A) Central memory CD4+T cell% of total CD4+T cells in branchial lymph nodes of different groups on day 13 after treatments start. N = 7 for control, MBTA, and rGL261 groups, respectively. N = 8 for the rGL261‐MBTA group. ns, *P*>0.05; One‐way ANOVA with multiple comparisons. B) Central memory CD8+T cell% of total CD8+T cells in branchial lymph nodes of different groups on day 13 after treatments start. N = 7 for control, MBTA, and rGL261 groups, respectively. N = 8 for the rGL261‐MBTA group. ns, *P*>0.05; *, *P*<0.05; ***, *P*<0.001; One‐way ANOVA with multiple comparisons. C) Percentage of GL261‐specific CD8+ T‐cells by MHCI‐tetramer staining in inguinal lymph nodes of old CR mice and control mice. N = 2 for each group. GL261 specific peptides for three tetramers: I: FSHHNIIRL, II: KVPRNQDWL, III: AALLNKLYA. D) Kaplan‐Meier survival curves of rechallenged old CR mice (n = 3) and control mice (n = 4). CR, complete regression. *P* = 0.0213, Log‐rank test.

Flow cytometry staining with adaptive immunity panel revealed increased CD44+CD62L+ central memory CD8+ T cells [(31.6±2.95)% versus (26.77±3.27)%] in branchial LNs of rGL261‐MBTA treated mice, compared to control group, on day 13 after the start of treatment (**Figure** **5**A,B). To confirm if the vaccinated mice develop tumor‐specific immunity against GL261 tumor cells, we quantified GL261 tumor antigen‐specific CD8+ T cells in two old rGL261‐MBTA mice that achieved CR and two age‐matched wild‐type mice. Three MHC class I‐tetramers loaded with different peptides (I: FSHHNIIRL,^[^ ^26^ ^]^ II: KVPRNQDWL,^[^ ^27^ ^]^ III: AALLNKLYA^[^ ^28^ ^]^) were designed for this purpose. We confirmed the generation of a higher number of MHC I‐Tetramer+ CD8+ T cells in LNs of rGL261‐MBTA vaccinated mice compared to age‐matched control mice (Figure 5C), indicating that rGL261‐MBTA vaccination established adaptive immunity against tumor‐specific antigens. Fourteen months after the last rGL261‐MBTA vaccine treatment, we intracranially rechallenged three rGL261‐MBTA mice that achieved CR with GL261 tumor cells. All rechallenged animals were free of GBM tumor development, while all four age‐matched control mice developed GBM tumors and died around 22 days after intracranial implantation (*P* = 0.0213, Log‐rank test) (Figure 5D). These data confirmed long‐term immunological memory against the same GBM cells in rGL261‐MBTA vaccinated mice.

### 2.6 rSB28‐MBTA Vaccine Significantly Prolongs Survival and may Eliminate GBM in The “Cold” SB28 GBM Model

**Figure 6:**
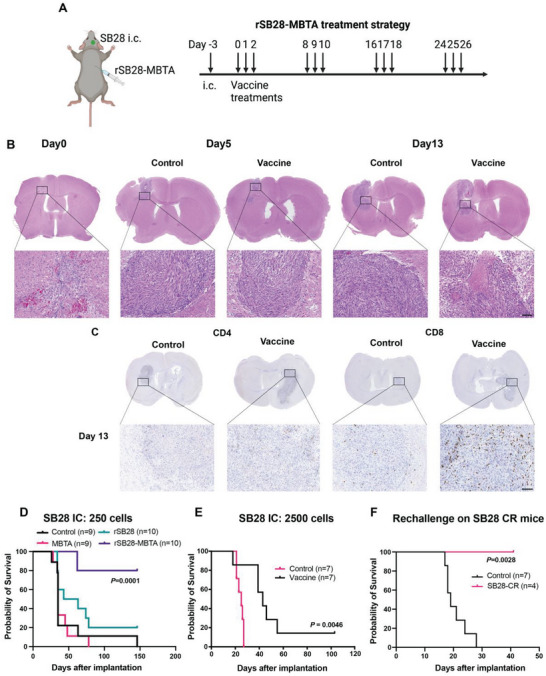
rSB28‐MBTA vaccine significantly prolongs survival of SB28 GBM model. A) Treatment strategy of rSB28‐MBTA vaccine. B) Histology of SB28 tumor tissues at different treatment time points. 2500 SB28 cells were implanted three days before the start of treatments. Solid tumors were observed on day 0, the start day of the vaccination. Vaccine, rSB28‐MBTA, the same as below. C) Representative CD4 and CD8 IHC staining of control and vaccinated tumors on day 13. Scale bars, 100 µm. D) Kaplan‐Meier survival curves of different treatment groups. 250 SB28 cells were implanted. *P* = 0.0001, Log‐rank test. E) Kaplan‐Meier survival curves of the control and vaccine groups. 2500 SB28 cells were implanted. *P* = 0.0046, Log‐rank test. F) Kaplan’’’‐Meier survival curves of rechallenged SB28‐CR mice (n = 4) and control mice (n = 7). *P* = 0.0028, Log‐rank test.

Since the GL261 GBM model was reported to be immunogenically “hot” and responsive to checkpoint inhibitor immunotherapies,^[^ ^29^ ^]^ we then tested our vaccine in the SB28 GBM model, a poorly immunogenic and highly checkpoint inhibitor‐resistant tumor, which is more consistent with human GBM.^[^ ^29^, ^30^ ^]^ SB28 cells have been reported to develop GBM efficiently after intracranially implanting a small number of cells.^[^ ^29^ ^]^ In our first round of testing, we established SB28 GBM tumors by intracranially implanting 250 cells per mouse and treated the mice in the same way as the GL261 model (**Figure** **6**A). We confirmed that 250 SB28 cells successfully established GBM in all control mice. The median survival time is 35 days for both saline and MBTA groups. rSB28‐treated mice showed a median survival time of 53 days, indicating a slight efficacy with irradiated tumor cells alone. Mice in the rSB28‐MBTA vaccine treatment group demonstrated the most extended survival with eight of ten vaccine‐treated mice surviving without GBM symptoms at day 146 after tumor cell implantation (Figure 6D, *P* = 0.0001, Log‐rank test).

We then tested our vaccine strategy in SB28 models with higher tumor burden (2500 cells). Two out of seven vaccinated mice achieved CR while none did in the control group. The survival time of the vaccine group was also significantly longer than the control group (median survival: 43 versus 25 days, *P* = 0.0046, Log‐rank test) (Figure 6E). We also compared the histology in control and vaccine groups at different time points (Figure 6B). On day 0 of vaccination (three days after implantation), a small solid tumor already developed in the brain. On day 5 (three days after the first treatment cycle), the SB28 tumors grew bigger without obvious differences between the control and vaccine groups. However, on day 13 (three days after the 2nd treatment cycle), we observed necrosis and more lymphocytes in the vaccinated SB28 tumors (Figure 6B). We also confirmed the increased T cells by CD4 and CD8 IHC staining. Few CD4+ and CD8+ T cells were found in the control SB28 tumors, but both were significantly increased in the vaccinated group (Figure 6C). We noticed that most CD4+ and CD8+ T cells were distributed in the peripheral SB28 tumor area with only a few T cells found in the center area, which is different from the GL261 model. Increasing T cell infiltration into GBM tissues could be a future direction to further enhance the vaccine efficacy.

We also confirmed immune memory against SB28 tumors in the vaccinated mice through tumor rechallenge. Four mice with CR in the rSB28‐MBTA vaccine group were rechallenged with SB28 cells four months after initial tumor cell implantation. None of the vaccinated mice developed GBM upon rechallenge, while all seven control mice developed GBM tumors with a median survival of 19 days after intracranial implantation (Figure 6F, *P* = 0.0028, Log‐rank test).

**Figure 7:**
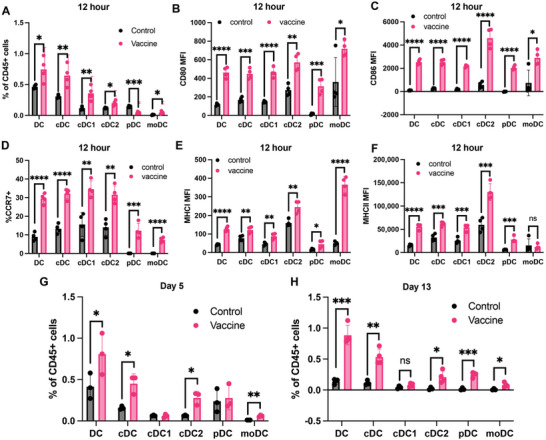
rSB28‐MBTA vaccine activated dendritic cells in lymph nodes. A) The percentage of each DC subtype in the vaccine‐draining lymph nodes 12 hours after the first vaccination. Vaccine, rSB28‐MBTA, the same as below. N = 4 for each group. B–F) CD80 (B), CD86 (C), CCR7+% (D), MHC I (E), and MHC II (F) were all increased in each DC subtype at 12 hours after vaccination. N = 4 for each group. G,H) The percentage of each DC subtype in the draining lymph nodes on Day 5 (G) and Day 13 (H) after the first vaccination. N = 3 for both groups on Day 5. N = 3 for the control group and N = 4 for the vaccine group on Day 13. ns, *P*>0.05; *, *P*<0.05; **, *P*<0.01; ***, *P*<0.001; ****, *P*<0.0001; unpaired t test.

We next examined the rSB28‐MBTA vaccine effects on dendritic cell subsets at different time points. At 12 h after the first vaccination, we observed significantly increased cDCs in the ILN (vaccine‐draining LN) [(0.65±0.17)% versus (0.31±0.03)% in the control group) (**Figure** **7**A). Among them, cDC1 [(0.36±0.12)% versus (0.12±0.03)%] increased more than cDC2 [(0.2±0.04)% versus (0.12±0.01)] cells. Moreover, immuno‐costimulatory signals (CD80 and CD86), migratory marker CCR7, and MHC I were significantly increased in DCs and all the DC subsets (cDC including cDC1 and cDC2, moDC, pDC) (Figure 7B–E). MHC II was also increased in DC subsets except in moDC (Figure 7F). On day 5 and day 13, two days after the first and second treatment cycles respectively, both ILN and spleens were enlarged in the rSB28‐MBTA group (Figure S6, Supporting Information). On both days, we did not observe significant changes in the cDC1 percentage of total CD45+ cells, but the cDC2 cell percentage in the vaccine group was much higher than in the control group (0.28% versus 0.06% on day 5, 0.21% versus 0.02% on day 13) (Figure 7G,H). In the SB28 tumors, we did not observe significant changes in the DC percentage, immune‐costimulatory markers, or MHC molecules on day 13 (Figure S7A–E, Supporting Information), which is different from the GL261 model. However, we also observed significantly increased PDL1 in the M2 macrophages of the vaccine group (Figure S7F,G, Supporting Information), which is consistent with the GL261 model.

Regarding the adaptive immune responses after vaccination, we confirmed vaccine‐induced cytotoxicity against SB28 cells in the vaccine‐draining LN by co‐culture assay (Figure S8A, Supporting Information). TIL analyses on day 13 revealed decreased SB28 cells (18.1% versus 28.6%), increased CD4+ T (4.76% versus 1.51%) and CD8+ T (4.7% versus 1.47%), and decreased Treg cells (13.56% versus 24.55%) in the vaccine group (Figure S8B–E, Supporting Information). Consistent with the GL261 model, we did not observe significant changes in the T cell exhaustion markers except for a trend of increased TIM3 in the CD8+ T cells (Figure S8F,G, Supporting Information).

### 2.7 rWTC‐MBTA Vaccine did not cause Severe Toxic Side Effects in Mice

**Figure 8:**
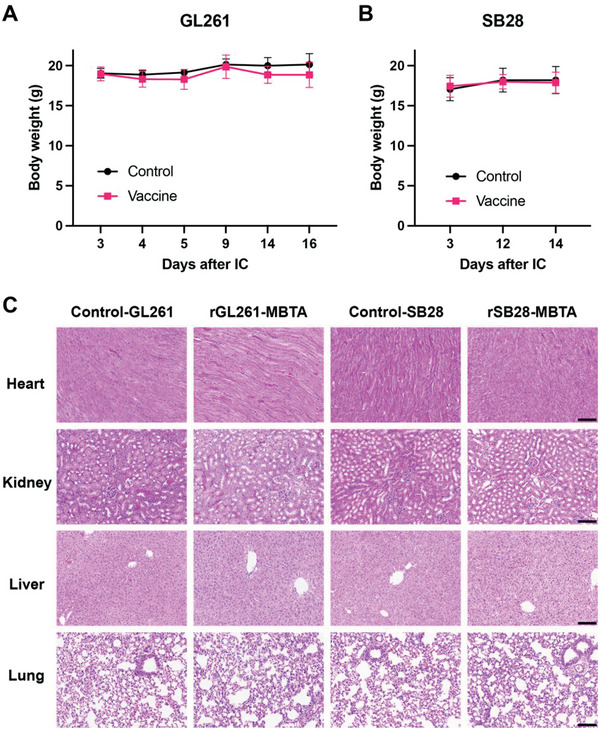
rWTC‐MBTA vaccine does not affect body weight and major organs, indicating low toxicity. (A‐B) The body weight was not affected in the rGL261‐MBTA A) and rSB28‐MBTA B) vaccination groups. N = 7 for the control and vaccine groups of the GL261 model, respectively. N = 15 for the control group and N = 16 for the vaccine group of the SB28 model. C) Representative H&E staining of the major organs in the control and vaccination groups.

We also examined the adverse events of our vaccine strategy in both GL261 and SB28 GBM animal models. We noticed a subcutaneous solid mass at the vaccine injection site and a small dry ulceration spot on the skin, which would disappear after vaccination cycles. We also checked the body weight and histology of the major organs. There was no significant weight loss in the vaccine groups (rGL261‐MBTA and rSB28‐MBTA), and we did not notice any apparent abnormalities in the major tissues of the vaccine mice (**Figure** **8**).

## 3 Discussion

Enhancing T‐cell priming and suppressing immune co‐inhibitory signals with reinforcement of costimulatory signals is an attractive strategy to convert immunologically “cold” tumors to “hot”. Despite the promise of immune checkpoint inhibitors upregulating in solid tumors, PD‐1/PD‐L1‐targeting checkpoint inhibitors and adoptive T‐cell transfer were often rendered ineffective against immunologically “cold” tumors like GBM. One of the significant obstacles to CAR‐T therapy for the treatment of GBM is the tumor's high heterogeneity preventing the targeting of all clonal populations.^[^ ^31^ ^]^ Other strategies that inhibit specific immunosuppressive signals in TME have not shown promising results in brain tumor patients either, suggesting that targeting immunosuppression alone is insufficient to enhance immunotherapy efficacy against GBM.^[^ ^7^ ^]^ In this study, we developed an autologous immunotherapeutic platform to overcome GBM tumor heterogeneity and therapeutic resistance.

In the present study, we evaluated the immunotherapeutic potential of the autologous cancer vaccine rWTC‐MBTA to trigger a potent antitumor response in vivo using orthotopic syngeneic mouse GBM preclinical models. The vaccination with rWTC‐MBTA successfully increased the survival of the tumor‐bearing mice, with some mice achieving CR. Our data suggested that the rWTC‐MBTA vaccination strategy activated DCs and the subsequent adaptive immune responses to counteract the tumor. We demonstrated that in the GL261 GBM animal model, rGL261‐MBTA required CD4+ and CD8+ T cells for vaccine efficacy, which was likely augmented by the activated DC populations in vaccine‐draining LNs. Additionally, the vaccine induced enduring immunological memory against GBM cells, demonstrated by the prevention of tumor development upon rechallenge.

Both MBTA and irradiated whole tumor cells are indispensable for the anti‐tumor efficacy in the rWTC‐MBTA vaccination strategy. Compared to the rWTC‐MBTA group, MBTA alone exhibited similar effects in enhancing the trafficking of APCs to the local draining LNs (Figure S1, Supporting Information). The MBTA group also showed comparable functional signatures (IFNγ+TNFα+, GranzymeB+) in the CD4+ and CD8+ T cells upon PMA activation (Figure 3). However, these activated T cells are not expected to be tumor‐specific because the activated DCs were not able to present tumor antigens to the naïve T cells without the combination with the irradiated tumor cells (source of the tumor antigens). It is most likely that the activated DCs in the MBTA alone group result from the immune‐activating effects of Mannan, TLR agonists and CD40 agonistic antibody,^[^ ^32^, ^33^, ^34^ ^]^ leading to their migration to the draining LN where they activate T cells that are not specific to the tumor. Accordingly, the MBTA alone group did not show any increased survival compared to the control group in both GL261 and SB28 GBM models (Figure 1B and Figure 6D). On the other hand, irradiated tumor cells were reported to cause weak immunogenicity but showed limited clinical impact.^[^ ^12^ ^]^ Consistently, rGL261 alone led to weaker cytotoxic signatures upon PMA activation than the MBTA or rGL261‐MBTA vaccine group (Figure 3). Minor efficacy was also observed in the irradiated tumor cell alone group in both GL261 and SB28 models (Figure 1B and Figure 6D). These results indicate that without the adjuvants (MBTA), dendritic cells were not able to efficiently deliver tumor antigens to the naïve T cells in the irradiated tumor cell alone group.

The dynamic change of DC subsets (Figure 7) after rSB28‐MBTA vaccination is most likely attributed to the different effects of the vaccine's components. Our vaccination consists of mannan‐BAM‐anchored irradiated whole tumor cells, Toll‐like receptor (TLR) agonists [lipoteichoic acid (LTA), polyinosinic‐polycytidylic acid (poly (I:C)), and resiquimod (R‐848)], and anti‐CD40 monoclonal antibody. Mannan, as a PAMP, can be recognized by the pattern recognition receptors and C‐type lectin receptors on DCs and other innate immune cells, and induces phenotypic and functional maturation of mouse DCs in a TLR4‐dependent manner.^[^ ^32^ ^]^ cDCs are the major DC subsets to prime naïve T cells and restimulate memory T cells in secondary lymphoid tissues (e.g., LN). Since TLR4 is generally expressed across cDCs,^[^ ^35^ ^]^ mannan is expected to induce maturation of both cDC1 and cDC2. TLR3 is highly expressed in both mouse and human cDC1 and promotes cDC1‐mediated cross priming of CD8+ T cells.^[^ ^19^, ^36^ ^]^ Poly (I:C), as a TLR3 agonist, activates cDC1 via TLR3. On the other hand, R‐848 activates cDC2 via TLR7, which is uniquely expressed in mouse and human cDC2. Notably, moDC and pDC were also activated by the rGL261‐MBTA and rSB28‐MBTA vaccines (Figure 2 and Figure 7), likely through TLR4 and TLR7, respectively.^[^ ^35^, ^37^, ^38^ ^]^ The contribution of moDC and pDC to our vaccine‐induced anti‐tumor immunity warrants further investigation.

The immunosuppressive TME is always a significant challenge to any ongoing immunotherapy trials against GBM. In this study, our vaccine revealed mixed effects on different aspects of the GBM TME: 1) We confirmed decreased regulatory T cells in the vaccinated mice in both GL261 and SB28 models (Figure 4C; Figure S8E, Supporting Information). 2) We quantified the MDSCs in the GL261 tumors and did not notice significant changes in the M‐MDSC or PMN‐MDSC in the vaccine group compared to the control group (Figure S5A–C, Supporting Information). 3) We quantified M2 macrophages in both GL261 and SB28 GBM tumors and did not notice significant changes in the cell percentage after vaccination. Instead, PDL1 expression was elevated in the M2 macrophages of the vaccine group in both models (Figure S5D,E, and Figure S7F,G, Supporting Information), which is consistent with DC vaccine treatment.^[^ ^39^ ^]^ DC vaccine treatments expanded a PDL1‐expressing tumor‐infiltrating myeloid cell population via IFNγ, and a significant survival benefit of the GL261 GBM model was obtained in DC vaccination when combined with anti‐PD1 monoclonal antibody blockade and/or a colony‐stimulating factor 1 receptor inhibitor.^[^ ^39^ ^]^ 4) We did not observe significant changes in the T cell exhaustion markers in the GBM tumors of the vaccinated mice, except for increased TIM3 expression in CD8+ T cells in both GL261 and SB28 models (Figure S4 and S8, Supporting Information). 5) The increased CD4+ and CD8+ T cells are mainly distributed to the peripheral area of SB28 tumors in the vaccine group, indicating that lymphocyte infiltration is also a big obstacle for immunotherapies in “cold” GBMs like the SB28 model. Notably, we noticed that rGL261 alone induced more exhausted CD8+ T cells and more PMN‐MDSC in the tumor tissues (Figure S4B and S5B, Supporting Information), indicating that the irradiated tumor cells alone induced more immunosuppressive signals in the brain tumors. However, when combined with MBTA in the rGL261‐MBTA vaccine group, such immunosuppressive signals were mostly reverted (Figure S4 and S5, Supporting Information). We hypothesize that the MBTA‐induced systemic pro‐inflammation responses were able to inhibit irradiated tumor cell‐induced T‐cell exhaustion and MDSC accumulation in the brain tumors, ensuring better efficacy in the rWTC‐MBTA vaccine group. Taken together, this study warrants further investigations of the potential combinations with anti‐TIM3, anti‐PD1/PDL1, and other immunotherapies to further enhance our vaccine efficacy.

Our study provides robust support for the translational potential of the rWTC‐MBTA vaccine strategy in clinical applications. In the context of patients with GBM, the surgical removal of tumor tissues enables the utilization of dissociated tumor cells to generate the rWTC‐MBTA vaccine. Administrating the rWTC‐MBTA vaccine back to the patients, particularly when addressing residual or recurrent tumors, holds significant promise. This is amplified by the limited tumor burden and the potential disruption of the TME in the remaining GBM tissue following surgery, enhancing the likelihood of more effective eradication. These factors collectively enhance the suitability and efficacy of the rWTC‐MBTA vaccine. Furthermore, grounded in the common principle of our vaccine, this approach has the potential for broader application to other tumor types.

While our study highlights the promising potential of the rWTC‐MBTA vaccine strategy for clinical application, it is essential to acknowledge certain limitations. First, the GBM preclinical models employed may not fully represent the diverse landscape of human gliomas. Enhancing the comprehensiveness of the study could involve incorporating a broader range of preclinical models that account for various glioma subtypes and disease scopes, including those with more tumor cell penetrations. Second, the potential interruption of the blood‐brain barrier at the intracranial implantation site should be taken into consideration, as it may impact the vaccine's interaction with the TME. Future studies could benefit from utilizing transgenic GBM models to circumvent the issue, providing a more accurate representation of the vaccine's efficacy in the complex intracranial environment.

In summary, our studies with the rWTC‐MBTA vaccine showed significant anti‐tumor immune response against GBM mouse models. The demonstrated efficacy of the rWTC‐MBTA vaccine may allow us to circumvent the labor‐intensive and expensive sequencing required by therapies targeting tumor‐specific epitopes. We foresee using the rWTC‐MBTA vaccine and similar strategies in combination with other immune‐modulating therapies to enhance the vaccine response and extend the current overall survival of patients with GBM.

## 4 Experimental Section

### Cell‐Lines

The SB28 cell line was kindly provided by Dr. Hideho Okada in UCSF under approved MTA. GL261 and SB28 cells were cultured in a complete DMEM medium (Gibco) containing 10% (vol/vol) FBS, 100 U mL^−1^ penicillin, and 100 µg mL^−1^ streptomycin (Gibco). The cell lines were tested negative for mycoplasma contamination using MycoAlert Mycoplasma Detection Kit (Lonza Inc, LT07‐218).

### Syngeneic GBM Models

All animal procedures reported in this study performed by NCI‐CCR affiliated staff were approved by the NCI Animal Care and Use Committee (ACUC) and by federal regulatory requirements and standards. All components of the intramural NIH ACU program were accredited by AAALAC International. C57BL/6 (6–8‐week‐old) mice were purchased from Charles River Laboratory. To establish GBM tumor models, female C57BL/6 mice were intracranially (1 mm rostral of the bregma, 2 mm right of midline, and 2 mm deep from the skull surface) inoculated with GL261 cells (50000) or SB28 (250 or 2500) cells in 2 µL Hank's Balanced Salt Solution (HBSS; Crystalgen). Tumor‐bearing mice were monitored every three days in the beginning and then daily after two weeks. Survival endpoints were defined as when any of the following criteria were reached: 1) a loss of more than 15% of initial body weight, 2) tumor‐protruding skull, 3) severe hunched posture and impaired mobility, 4) ataxia.

For the T‐cell depletion studies, mice were injected with 250 µg anti‐CD4 antibody (clone GK1.5; BioXcell) and/or 250 µg anti‐CD8 antibody (clone 53–6.7; BioXcell) on Day −2, −1 day, and on the day of intracranial injection of GL261 cells, and weekly after that.

For tumor rechallenge studies, mice that achieved complete regression of GL261 or SB28 tumors and naïve female C57BL/6 mice were inoculated with 50000 GL261 cells or 2500 SB28 cells in 2 µL HBSS solution in the left frontal lobe (1 mm rostral of the bregma, 2 mm left of midline, and 2 mm deep from the skull surface).

### rWTC‐MBTA Vaccination

Mannan‐BAM synthesis was performed as previously reported.^[^ ^40^ ^]^ For rWTC‐MBTA vaccine preparation, tumor cells in culture were collected and resuspended in PBS at a density of 1 million cells per 50 µL (dose per mouse) and irradiated with 100 Gy in a ^137^Cs MARK I model irradiator (JL Shepherd & Associates, San Fernando, CA). After irradiation, 1 million irradiated tumor cells in 50 µL PBS were mixed with 50 µL 0.2 mM mannan‐BAM solution containing 25 µg LTA, 25 µg poly (I:C), 25 µg R‐848 (HCl form), and 1 µg anti‐CD40 (BioXcell, BE0016‐2) (single injection dose per mouse) for 0.5‐1 h, and then injected subcutaneously to the right flank of treated animals.

### Tissue Dissociation

LNs, spleens, and GBM tissues were isolated at the indicated time points mentioned in the text. LNs and spleens were manually ground and filtered through MACS SmartStrainer (70 µm) (Miltenyi Biotec, Inc.) before staining. GBM tissues were dissociated with a mouse Tumor Dissociation Kit and a GentleMACS Dissociator (Miltenyi Biotec) according to the manufacturer's instructions. After dissociation, 500000 or a maximum number of cells (total cell number <500000) were used for subsequent flow cytometry staining. Erythrocytes were also lysed by 1xRBC lysis buffer after brain tumor dissociation.

### Co‐Culture Assay

LN or spleen immune cells were co‐cultured with GL261 or SB28 cells (30000‐50000 tumor cells) at a ratio of 10:1. After 48 h of co‐culture, the co‐cultured cells were incubated with anti‐CD45, anti‐CD3ε, anti‐CD4, anti‐CD8α, anti‐CD107a, anti‐GranzymeB, anti‐IFN‐γ and anti‐TNF‐α antibodies for immune functional analysis. Tumor cells were counted using CountBright Absolute Counting Beads (ThermoFisher Scientific).

### Flow Cytometry Staining

All flow cytometry staining panels were listed in the supplementary table with detailed antibody information. For each panel, about 500000 cells were used for flow cytometry staining. Surface marker staining was performed in flow staining buffer (PBS+2% FBS+1 mM EDTA+0.02% sodium azide) at four degrees for thirty minutes. Dead cells were labeled with a fixable live/dead dye (ThermoFisher Scientific, L23105) in PBS at room temperature for 15 min. eBioscience Foxp3 / Transcription Factor Staining Buffer Set (ThermoFisher Scientific, catalog No. 00‐5523‐00) was used for FOXP3 staining after cell surface marker staining. For Intracellular cytokine/Granzyme B staining, after surface marker and dead cell staining, cells were fixed (BD Biosciences, 554 655), permeabilized (ThermoFisher Scientific, catalog No. 00‐5523‐00), and finally stained with intracellular antibodies at four degrees for one hour. Stained cells were analyzed by flow cytometry (BD Biosciences, FACSymphony). Data analysis was performed using FlowJo software (TreeStar).

### The Immune Cell Populations Mentioned in the Text were Defined as follows

Dendritic cells (CD45+Dump‐CD11c+MHCII+ or CD45+Dump‐F4/80‐CD11c+MHCII+); Macrophages (CD45+Dump‐CD11c‐CD11b+Ly6G‐Ly6C‐/low); Monocytes (CD45+Dump‐CD11c‐CD11b+Ly6G‐Ly6Chigh); MHC class II+ Monocytes (CD45+Dump‐CD11c‐CD11b+Ly6G‐Ly6ChighMHCII+); CD4+ T cell (CD45+TCRβ+CD4+CD8‐ or CD45+CD3ε+CD4+CD8‐); CD8+ T cell (CD45+TCRβ+CD4‐CD8+ or CD45+CD3ε+CD4‐CD8+); Treg (CD45+TCRβ+CD4+CD8‐FOXP3+); cDC (CD45+Dump‐F4/80‐CD11c+MHCII+Ly6C‐); moDC (CD45+Dump‐F4/80‐CD11c+MHCII+Ly6C+CD11b+); pDC (CD45+Dump‐F4/80‐CD11c+MHCII+Ly6C+CD11b‐); cDC1 (CD45+Dump‐F4/80‐CD11c+MHCII+Ly6C‐SIRPα‐XCR1+); cDC2 (CD45+Dump‐F4/80‐CD11c+MHCII+Ly6C‐SIRPα+XCR1‐); M2 macrophage (CD45+Dump‐F4/80+CD206+); M‐MDSC (CD45+Dump‐CD11c‐CD11b+Ly6G‐Ly6ChighMHCII‐SSClow); PMN‐MDSC (CD45+Dump‐CD11c‐CD11b+Ly6G+Ly6C‐/lowMHCII‐SSChigh). Gating strategies for DC subtypes and MDSCs were presented in the supporting Figure S9 and S10 (Supporting Information), respectively.

### Statistical Analysis

The statistical analyses were performed using GraphPad Prism 8 for macOS. Sample size (n) was presented in the figures and/or figure legends. Kaplan‐Meier survival curves were analyzed by Log‐rank test. For other graphs, data was presented as mean ± SD, and the *P* values were calculated by unpaired t test (two‐tailed) or One‐way ANOVA with multiple comparisons.

## Conflict of Interest

The authors declare no conflict of interest.

## Supporting information

Supporting Information

## Data Availability

The data that support the findings of this study are available from the corresponding author upon reasonable request.;
