# Supplementary material for: rWTC‐MBTA Vaccine Induces Potent Adaptive Immune Responses Against Glioblastomas via Dynamic Activation of Dendritic Cells
Source: Adv Sci (Weinh). 2024 Jan 31;11(14):2308280. doi: 10.1002/advs.202308280 (PMC11005728; doi:10.1002/advs.202308280)
Supplement: Supplementary file 1 — Supporting Information [file ADVS-11-2308280-s001.pdf]

ADVANCED SCIENCE
Open Access

Supporting Information

for *Adv. Sci.*, DOI 10.1002/advs.202308280

rWTC-MBTA Vaccine Induces Potent Adaptive Immune Responses Against Glioblastomas via Dynamic Activation of Dendritic Cells

Herui Wang, Rogelio Medina, Juan Ye, Yaping Zhang, Samik Chakraborty, Alex Valenzuela, Ondrej Uher, Katerina Hadrava Vanova, Mitchell Sun, Xueyu Sang, Deric M. Park, Jan Zenka, Mark R. Gilbert, Karel Pacak and Zhengping Zhuang**

rWTC-MBTA VACCINE INDUCES POTENT ADAPTIVE IMMUNE RESPONSES AGAINST GLIOBLASTOMAS VIA DYNAMIC ACTIVATION OF DENDRITIC CELLS

Herui Wang*#1, Rogelio Medina*1, Juan Ye*1, Yaping Zhang[1], Samik Chakraborty[2], Alex Valenzuela[1], Ondrej Uher[3], Katerina Hadrava Vanova[3], Mitchell Sun[1], Xueyu Sang[1], Deric M. Park[4], Jan Zenka[5], Mark R. Gilbert[1], Karel Pacak[3], Zhengping Zhuang#1

Affiliations:

[1] Neuro-Oncology Branch, National Cancer Institute, National Institutes of Health, Bethesda, Maryland, United States.

[2] NE1 Inc., New York, NY 10022

[3] *Eunice Kennedy Shriver* National Institute of Child Health and Human Development, National Institutes of Health, Bethesda, Maryland, United States.

[4] John Theurer Cancer Center, HUMC, Hackensack Meridian School of Medicine

[5] Department of Medical Biology, Faculty of Science, University of South Bohemia, České Budějovice, Czech Republic

* These authors contributed equally

# Co-corresponding authors

Running title: rWTC-MBTA vaccine elicits adaptive immune responses in GBM

Corresponding authors:

Herui Wang, Ph.D.

Staff Scientist

Neuro-Oncology Branch

National Cancer Institute

Center for Cancer Research

National Institutes of Health

Building 37, Room 1004

37 Convent Dr.

Bethesda, MD 20892

herui.wang@nih.gov
Phone: 240-760-7677
Fax: 240-541-4523

Zhengping Zhuang, M.D., Ph.D.
Senior Investigator
Neuro-Oncology Branch
National Cancer Institute
Center for Cancer Research
National Institutes of Health
Building 37, Room 1000
37 Convent Dr.
Bethesda, MD 20892
zhengping.zhuang@nih.gov
Phone: 240-760-7055
Fax: 240-541-4523

Supplementary Figure and Figure legends

Supplementary Figure 1. rGL261-MBTA vaccine increased innate immune cells to the draining lymph nodes. %Dendritic cell (DC) (A), %total monocyte (B), %MHCII+ monocyte (C), and %macrophage (D) of total CD45+ cells in branchial lymph nodes of different treatment groups were summarized. N=10 for each group. ns, $P>0.05$; *, $P<0.05$; ***, $P<0.001$; ****, $P<0.0001$; One-way ANOVA with multiple comparisons.

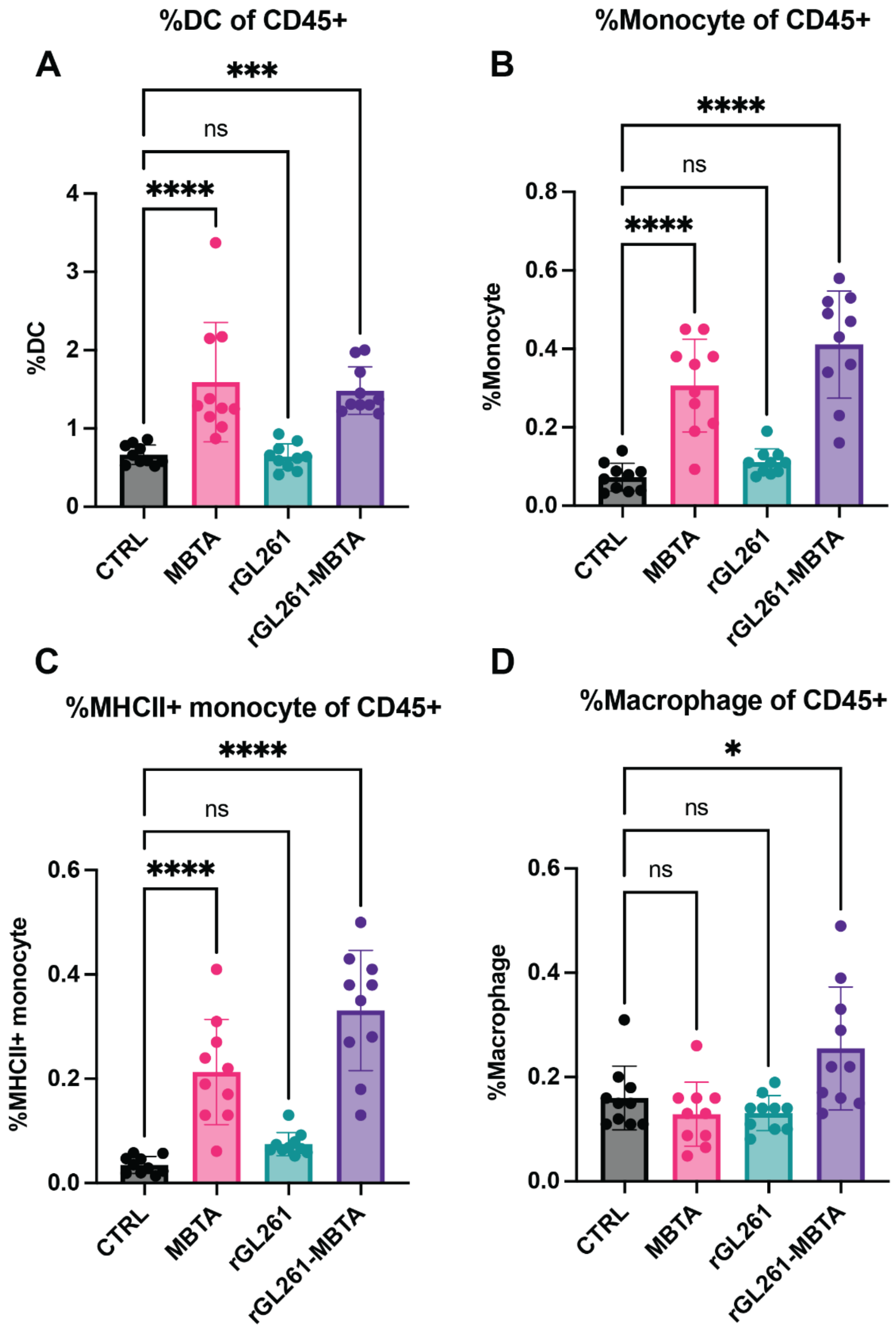

A
%DC of CD45+
***
ns
****
%DC
4
3
2
1
0
CTRL
MBTA
rGL261
rGL261-MBTA
B
%Monocyte of CD45+
****
ns
****
%Monocyte
0.8
0.6
0.4
0.2
0.0
CTRL
MBTA
rGL261
rGL261-MBTA
C
%MHCII+ monocyte of CD45+
****
ns
****
%MHCII+ monocyte
0.6
0.4
0.2
0.0
CTRL
MBTA
rGL261
rGL261-MBTA
D
%Macrophage of CD45+
*
ns
ns
%Macrophage
0.6
0.4
0.2
0.0
CTRL
MBTA
rGL261
rGL261-MBTA

Supplementary Figure 2. (A) Enlarged vaccine-draining lymph nodes and spleens in the rGL261-MBTA vaccinated mice on day 7. (B-C) The absolute number of DC subsets in the ILN (B) and spleen (C) of rGL261-MBTA vaccinated mice on day 13. N=4 for each group. *, *P*<0.05; **, *P*<0.01; ***, *P*<0.001; unpaired t test.

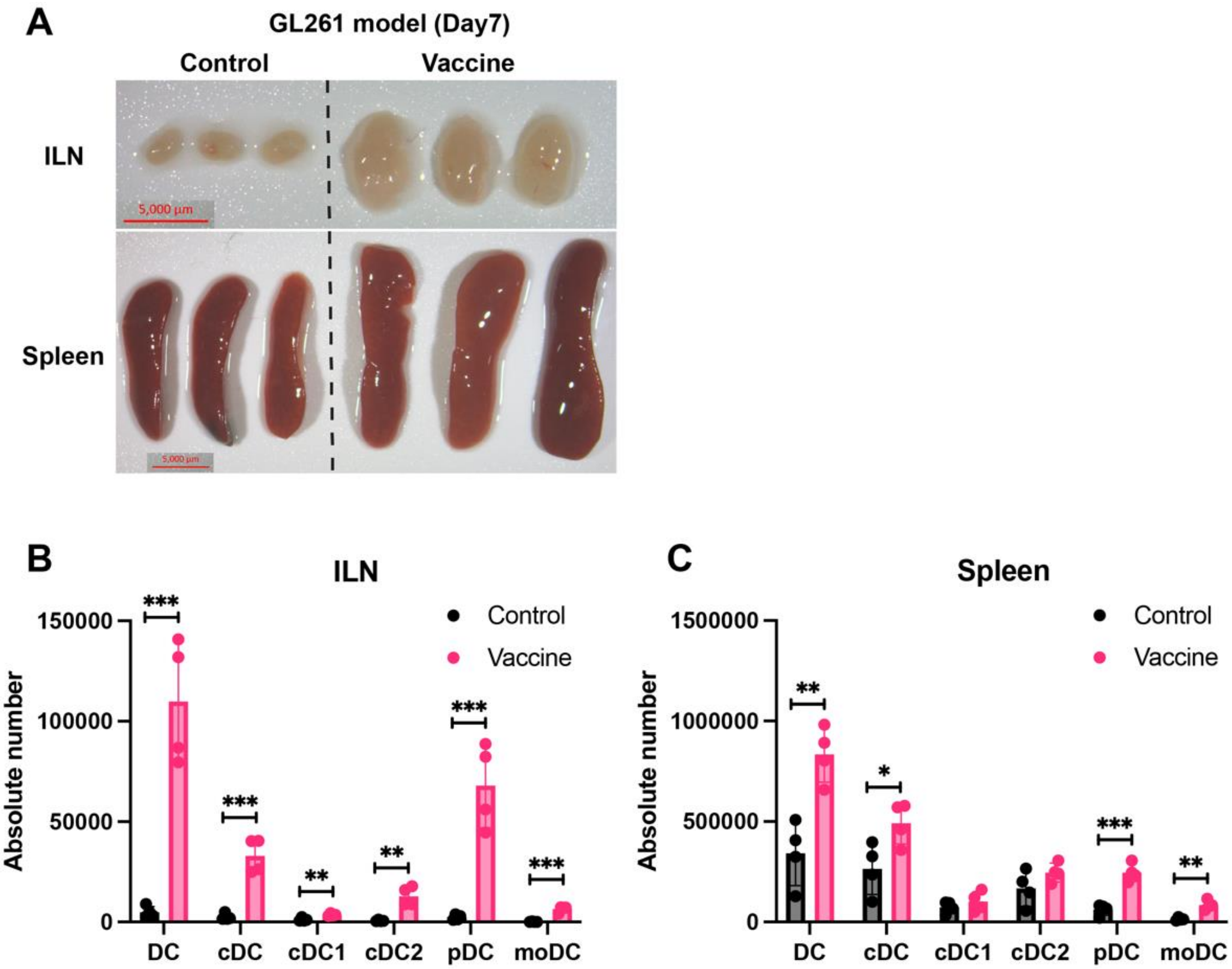

Supplementary Figure 3. Characterization of DC subtypes in GL261 brain tumors on Day 13. (A) Percentage of each DC subtype of the total live dissociated cells. No significant differences between the control and vaccine (rGL261-MBTA) groups. (B-E) MFI of CD80 (B), CD86 (C), MHC I (D), and MHC II (E) in each DC subtype. N=3 for the control group and N=4 for the vaccine group. *, $P<0.05$; **, $P<0.01$; unpaired t test.

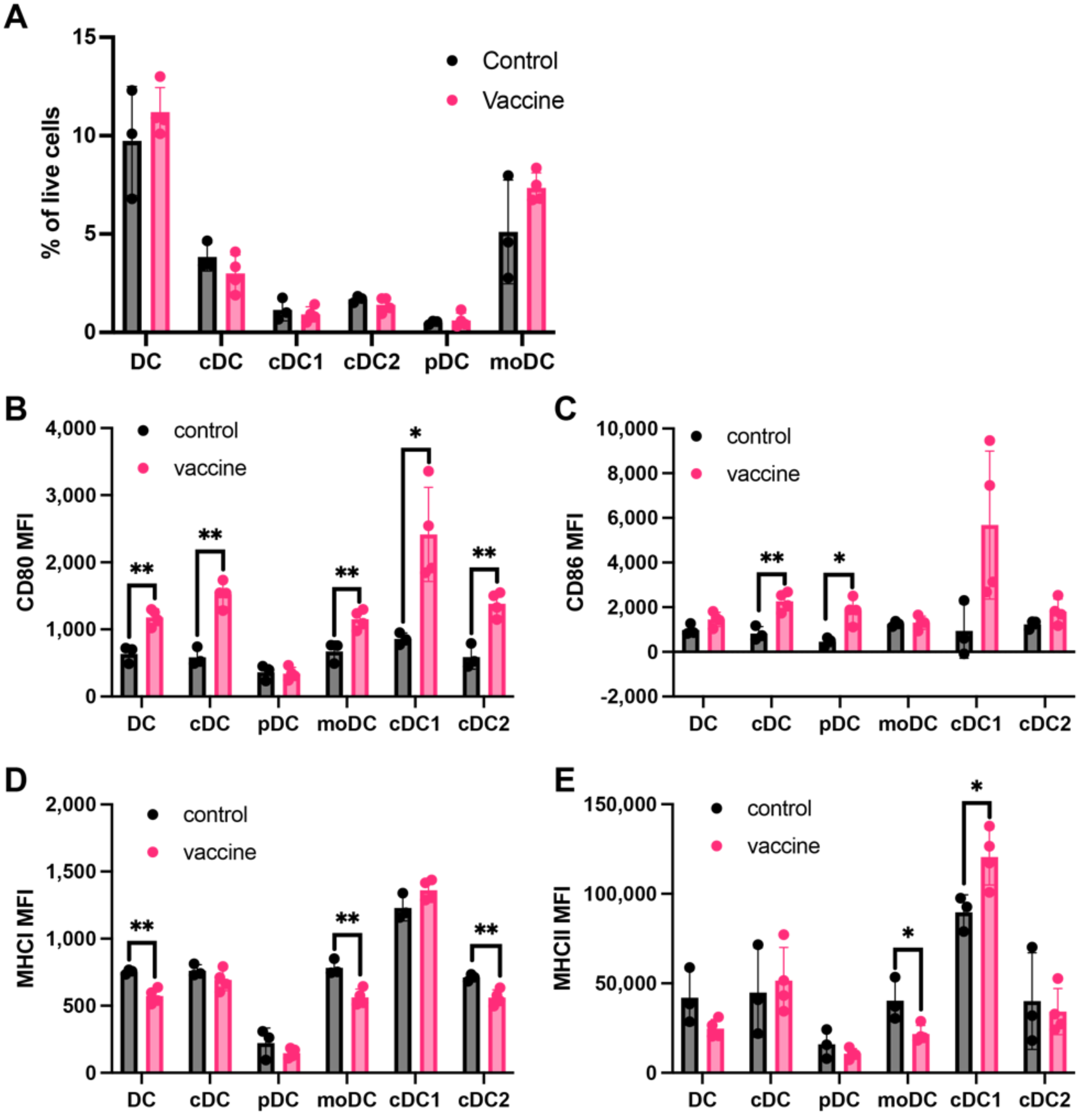

Supplementary Figure 4. Characterization of the exhaustion markers in CD4 (A) and CD8 (B) T cells of GL261 brain tumors on Day 13 from the start of vaccination. N=7 for each group. ns, *P*>0.05; *, *P*<0.05; **, *P*<0.01; ***, *P*<0.001; One-way ANOVA with multiple comparisons.

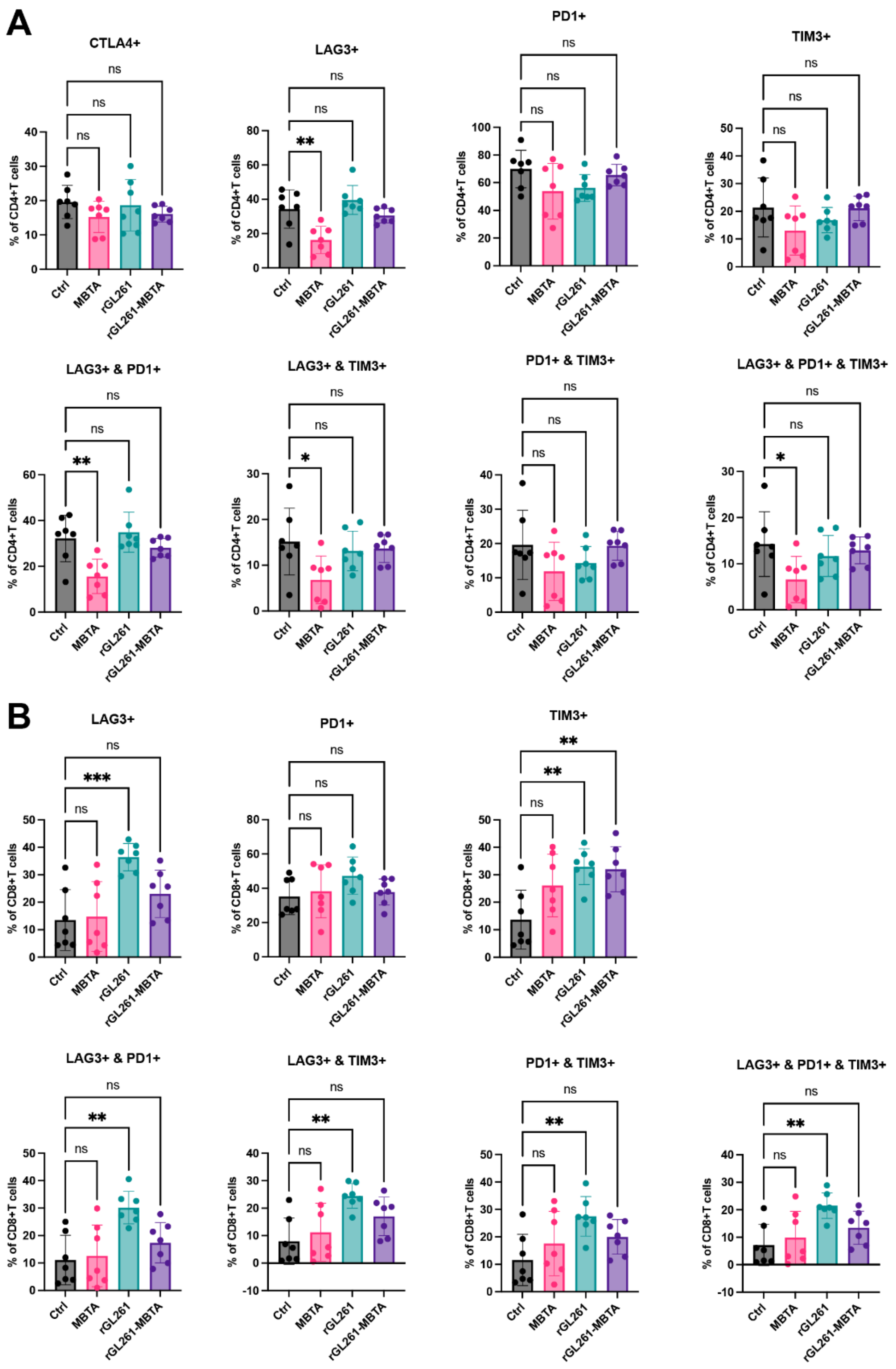

Supplementary Figure 5. Characterization of the immune suppressor cells in GL261 brain tumors. (A-C) %M-MDSC, %PMN-MDSC, and %total MDSC in brain tumors of each group. No significant difference was observed between the control and vaccine (rGL261-MBTA) groups. N=8 for the rGL261-MBTA group and N=7 for each of the other three groups. ns, *P*>0.05; *, *P*<0.05; **, *P*<0.01; ***, *P*<0.001; One-way ANOVA with multiple comparisons. (D) %M2 macrophages in control and vaccine groups. N=3 for the control group and N=4 for the vaccine (rGL261-MBTA) group. ns, *P*>0.05; unpaired t test. (E) PDL1 expression was elevated in M2 macrophages of vaccinated mice. N=3 for the control group and N=4 for the vaccine (rGL261-MBTA) group. **, *P*<0.01; unpaired t test.

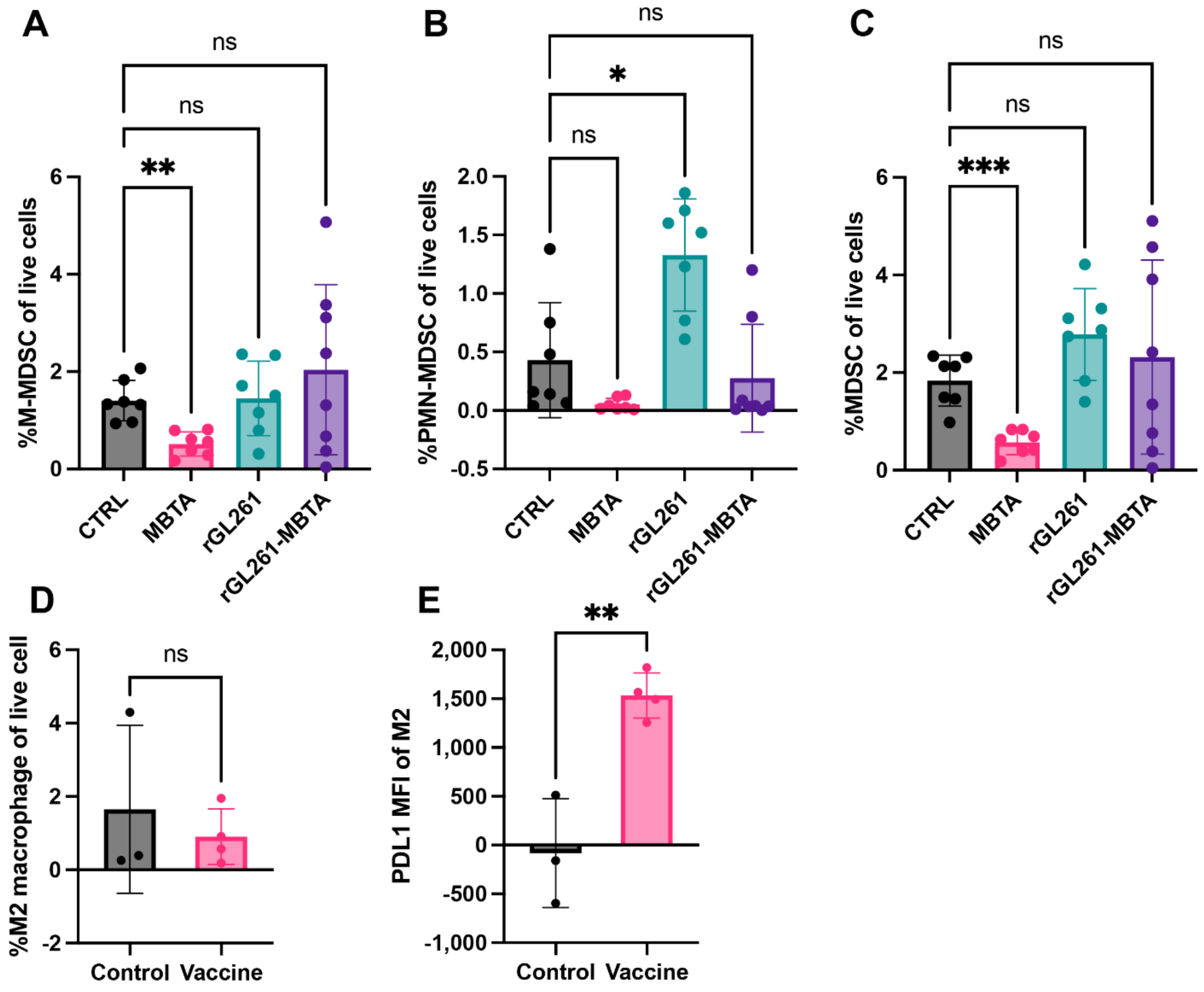

Supplementary Figure 6. Enlarged draining lymph nodes and spleens in the rSB28-MBTA vaccinated mice. Vaccine, rSB28-MBTA treatment group.

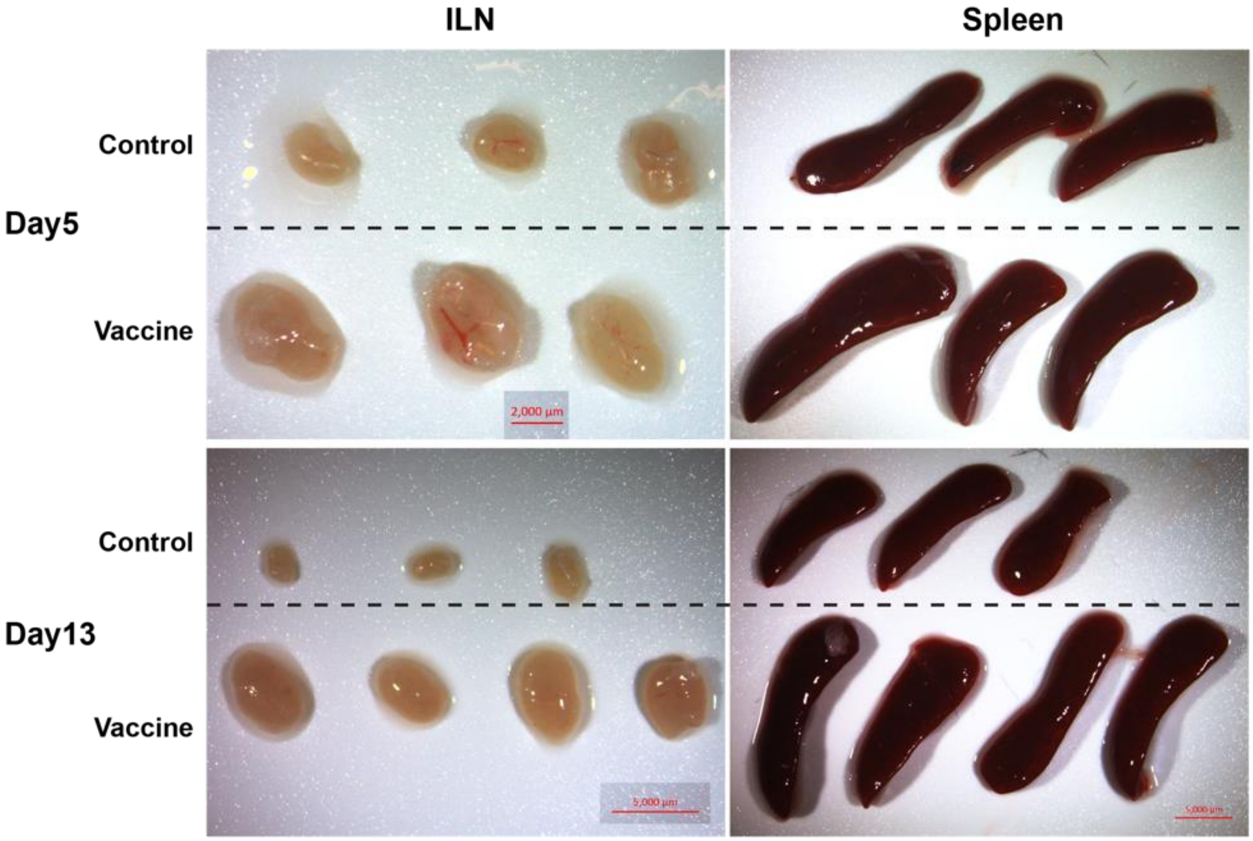

Supplementary Figure 7. Characterization of DC subtypes in SB28 brain tumors on Day 16 from the start of vaccination. (A) Percentage of each DC subtype of the total live dissociated cells. (B-E) MFI of CD80 (B), CD86 (C), MHC I (D), and MHC II (E) in each DC subtype. (F) Percentage of M2 macrophages of the total live dissociated cells. (G) MFI of PDL1 in M2 macrophages. N=4 for the control group and N=5 for the vaccine (rSB28-MBTA) group. ns, *P*>0.05; *, *P*<0.05; unpaired t test.

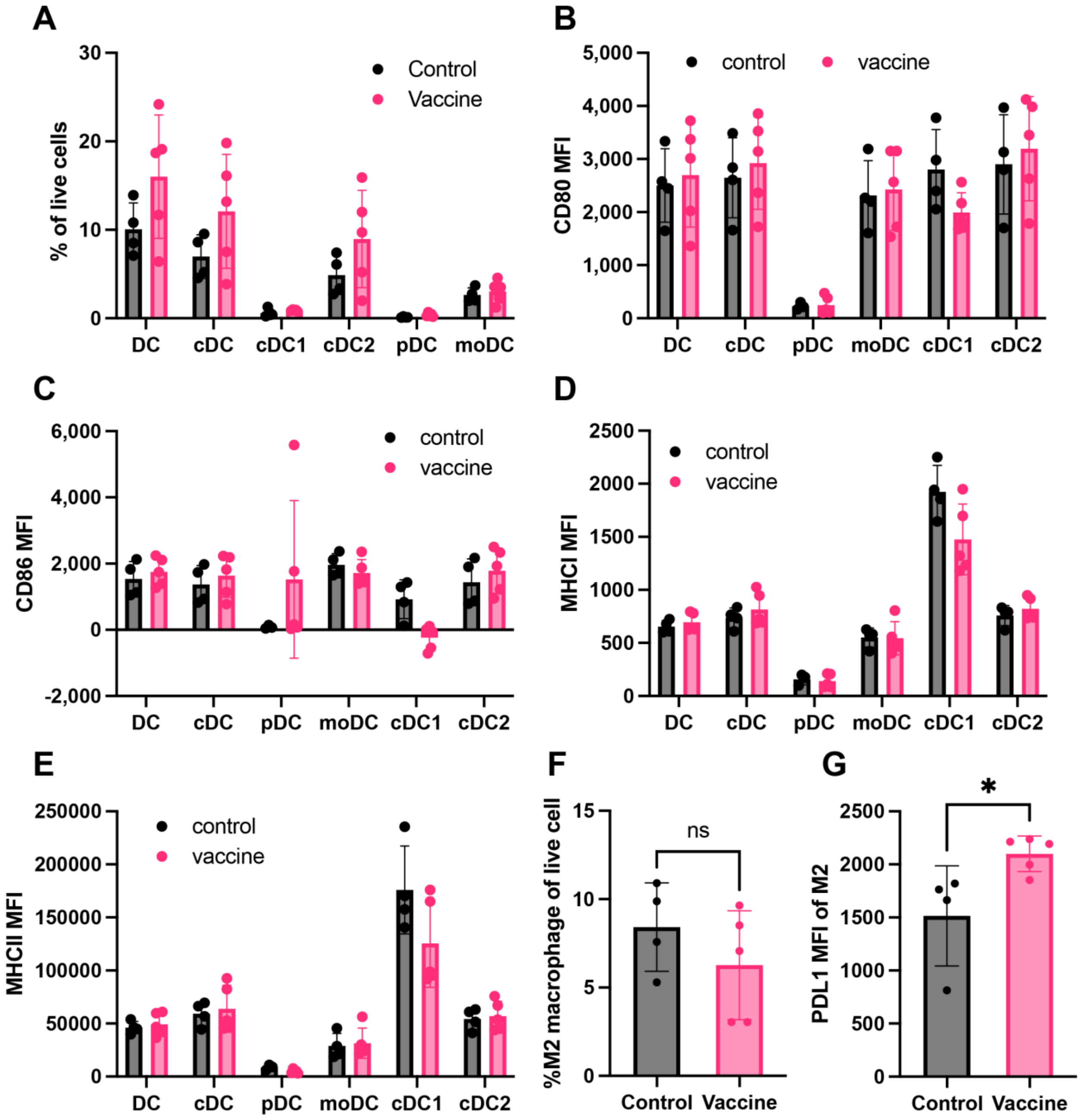

Supplementary Figure 8. Characterization of adaptive immune cells in rSB28-MBTA vaccinated mice. (A) Live SB28 tumor cell counts after co-culture with splenocytes of control and vaccine (rSB28-MBTA) mice. More SB28 tumor cells were killed by vaccinated splenocytes. N=5 for each group. (B) Less SB28 tumor cells in the dissociated tumor tissues of vaccinated mice. (C-E) CD4+T%, CD8+T%, and Treg% in control and vaccine groups. (F-G) Exhaustion markers in CD4+T and CD8+T cells. For B-G, N=4 for the control group and N=5 for the vaccine (rSB28-MBTA) group. ns, $P>0.05$; *, $P<0.05$; ***, $P<0.001$; ****, $P<0.0001$; unpaired t test.

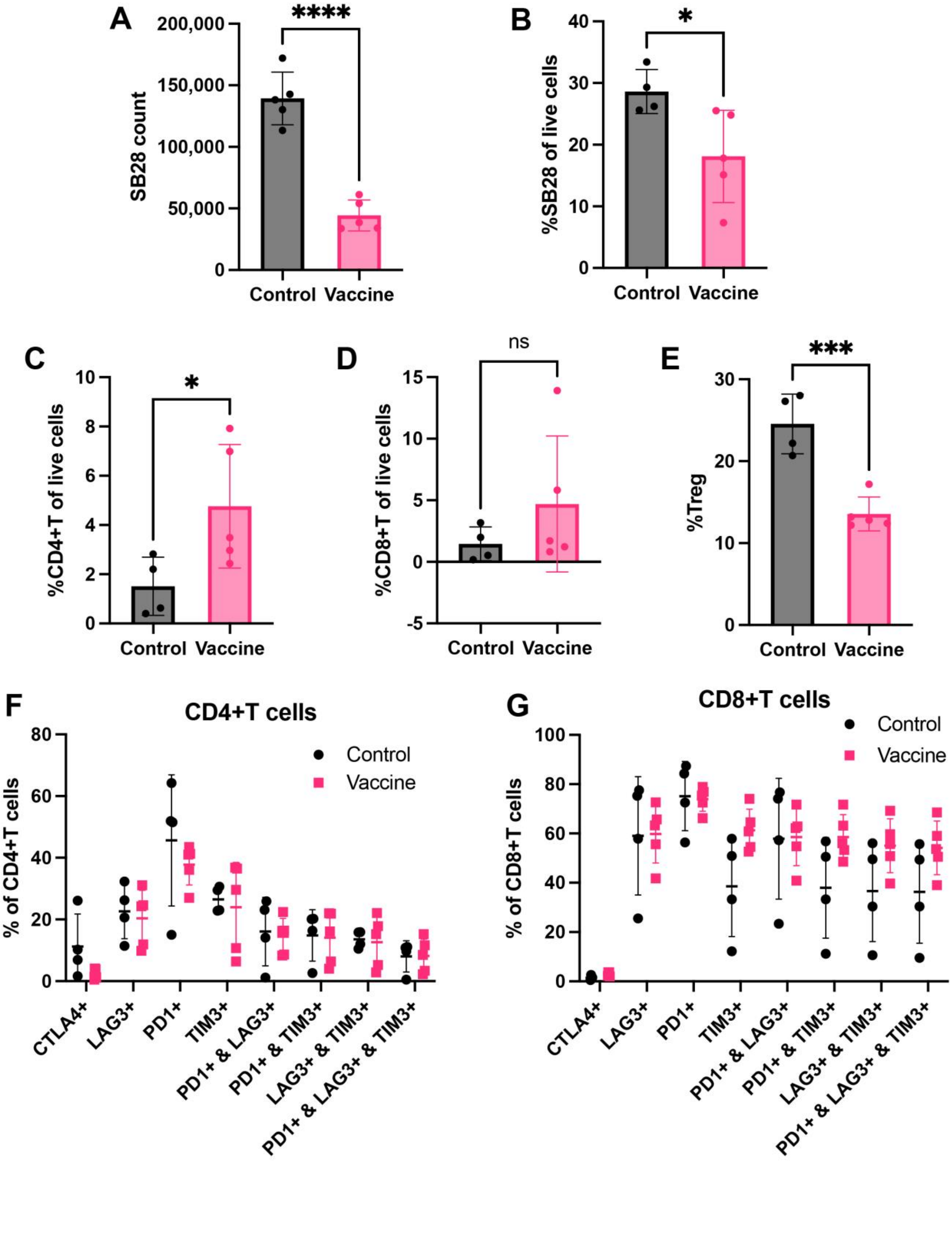
A
B
C
D
E
F
G
SB28 count
%SB28 of live cells
%CD4+T of live cells
%CD8+T of live cells
%Treg
****
*
*
ns
***
Control
Vaccine
CD4+T cells
CD8+T cells
% of CD4+T cells
% of CD8+T cells
CTLA4+
LAG3+
PD1+
TIM3+
PD1+ & LAG3+
PD1+ & TIM3+
LAG3+ & TIM3+
PD1+ & LAG3+ & TIM3+

Supplementary Figure 9. Gating strategy of DC subtypes.

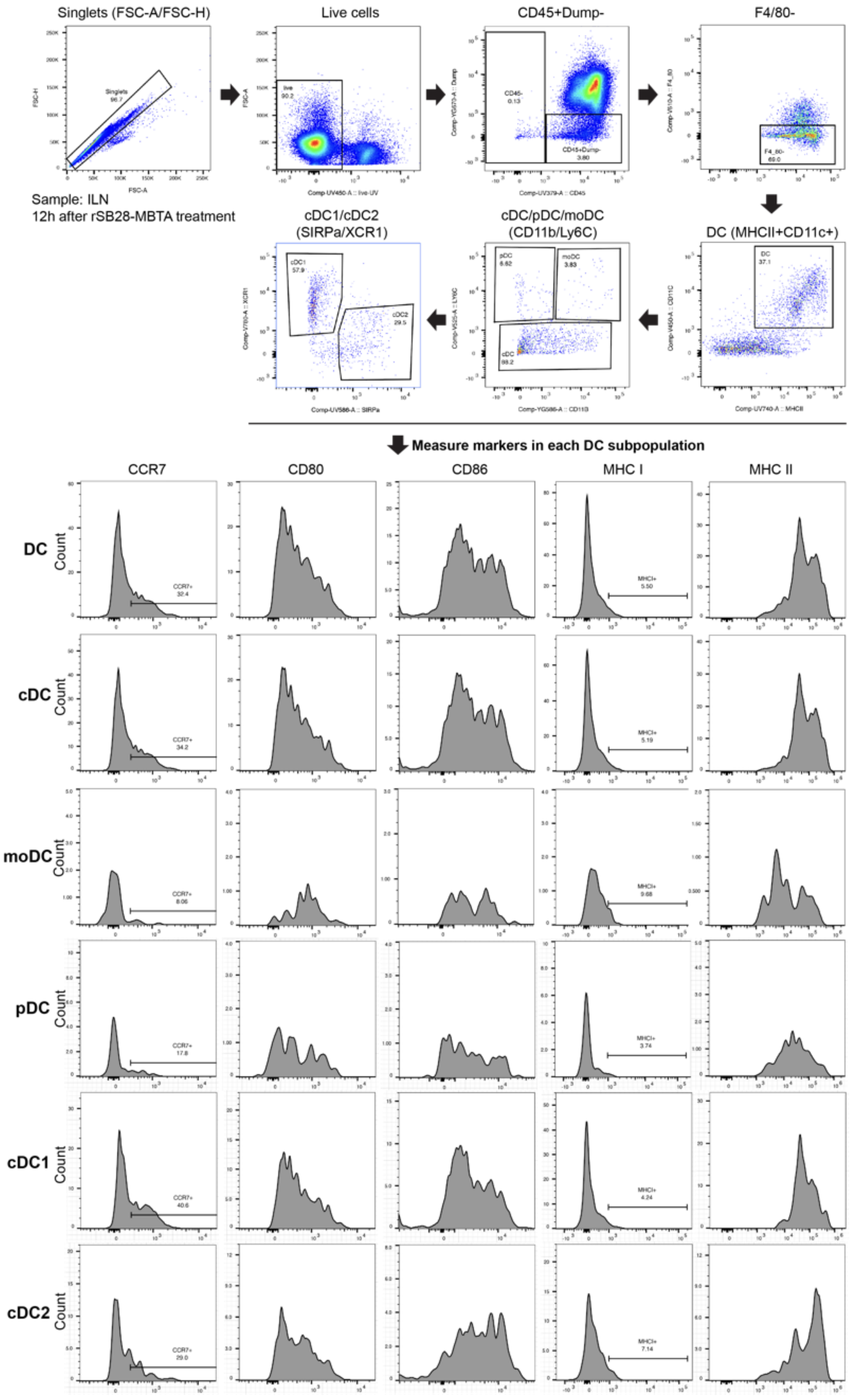

Supplementary Figure 10. Gating strategy of innate immune cells and MDSC.

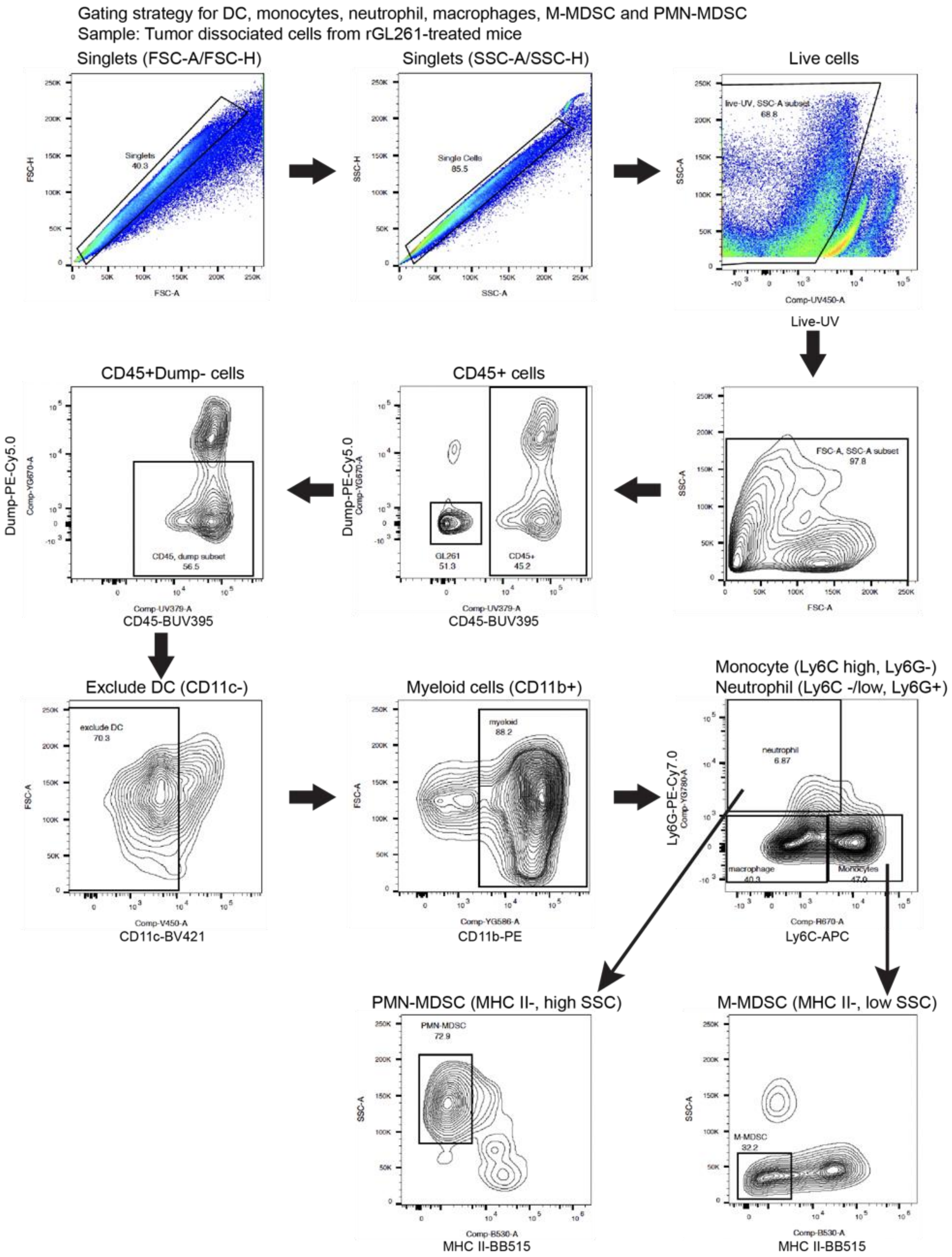

Supplementary Table. Flow staining panel used in this study. Provided in a separate Excel file.
